# Antitumor activity of 5-fluorouracil polymeric nanogel synthesized by gamma radiation on a rat model of colon carcinoma: a proposed mechanism

**DOI:** 10.1007/s12672-023-00733-z

**Published:** 2023-07-26

**Authors:** Omayma A. R. Abo-Zaid, Fatma S. M. Moawed, Wael E. M. Barakat, Mohamed Mohamady Ghobashy, Esraa S. A. Ahmed

**Affiliations:** 1 Biochemistry and Molecular Biology Department, Faculty of Vet. Med, Benha University, Benha, Egypt; 2 Health Radiation Research, National Center for Radiation Research and Technology, Egyptian Atomic Energy Authority, Nasr City, Cairo, 11787 Egypt; 3 Radiation Research of Polymer Chemistry Department, National Center for Radiation Research and Technology, Egyptian Atomic Energy Authority (EAEA), Cairo, Egypt; 4 Radiation Biology Research, National Center for Radiation Research and Technology, Egyptian Atomic Energy Authority, Cairo, Egypt

**Keywords:** 5-fluorouracil nanogel, Colon cancer, Autophagy, TLR2, NF-κβ, PI3K, AKT, mTOR

## Abstract

The use of 5-fluorouracil (5-FU) is associated with multifaceted challenges and poor pharmacokinetics. Accordingly, our study was designed to prepare 5-FU nanogel as a new form of the colon cancer chemotherapeutic drug 5-FU using polyacrylic acid and gelatin hybrid nanogel as efficient drug carriers. Alongside the in vivo chemotherapeutic evaluation, the anti-proliferative and anti-apoptotic efficacy were carried out for 5-FU nanogel against 1,2-dimethylhydrazine (DMH, 20 mg/kg) and γ-radiation (4 Gy)-prompted colon dysplasia in rats compared to 5-FU. The morphology and size of 5-FU nanogel were characterized by transmission electron microscopy (TEM) and dynamic light scattering (DLS) in addition to cytotoxicity assay. The expression of phosphoinositide-3-kinase (PI3K)/Akt, mammalian target of rapamycin (mTOR); Toll-like receptor2 (TLR2)/nuclear factor kappa B), adenosine monophosphate (AMP)-activated protein kinase (AMPK) and its downstream autophagy-related genes in addition to apoptotic markers were measured in colon tissues. Results: 5-FU nanogel reduced the levels of the TLR2/ NF-κβ as well as the expression of PI3K/AKT/mTOR. Moreover, it promoted autophagy through the activation of the AMPK and its downstream targets which consequently augmented the intrinsic and extrinsic apoptotic pathways. Conclusion: Collectively, these data might strengthen the therapeutic potential of 5-FU nanogel which can be used as an antitumor product for colon cancer.

**Supplementary Information:**

The online version contains supplementary material available at 10.1007/s12672-023-00733-z.

## Introduction

Globally, colorectal cancer is the second leading cause of death and the third most common cancer[1]. Due to westernization, the incidence of colorectal cancer is rising in developed as well as middle- and low-income nations. The majority of the rise in colorectal cancer cases can be attributed to unhealthy lifestyle choices, particularly acclimating to fast food, packaged foods, and inactivity [2]. Uncontrolled cell proliferation and growth of colonic crypt epithelial lining cells is a hallmark of colorectal cancer, which begins as hyperplasia and slowly progresses to invasive carcinoma [3]. Several animal models of colon cancer have been developed to investigate the molecular pathogenesis of the disease and the potential roles of various nutritional and pharmacologic preventatives [4]. The 1,2-dimethylhydrazine (DMH) model of the dysplastic colon is widely used among the various chemically induced animal models [5].

For both palliative and adjuvant colorectal cancer treatment, 5-FU is the first-line chemotherapeutic agent. However, the 5- FU may be rapidly leaching and have poor biostability in living organisms. Moreover, hepatic dihydropyrimidine dehydrogenase catabolizes more than 80% of 5-FU, while the remaining 20% causes non-selective action against healthy cells [6]. Additionally, multidrug resistance is among the most serious adverse effects and limitations of 5-FU [7].

Due to the serious adverse side effects of the chemotherapeutic agents, advanced methods for curing cancer, such as nanogel used to carry and release 5-fluorouracil to minimize side effects and optimized their pharmaceutic effect. Moreover, encapsulation or conjugation of 5-FU within nanoparticle drug delivery systems leads to high-performance issues due to distribution, bio-homogeneity, and biodegradation of the carrier moieties [8].

The advance of biodegradable nanogel with increasingly sophisticated functions rather than macrogol has greatly impacted biomedical applications to expand cancer treatment by drug delivery systems. Nanogels are hydrogels with in-vivo stability, internal networks for carrying bioactive molecules, a tunable porous structure, and a tiny particle size in the nanometre range, and a large surface area for biofunctionalization enhancing drug loading capacity making them ideal for drug delivery systems. Moreover, they can be designed to be stimulus-responsive and react to internal or external stimuli such as pH, and temperature, thus resulting in the controlled release of loaded drugs [9].

Polymers are used extensively in drug delivery systems due to their high stability, solubility, drug-loading capacity, and selective targeting potential [10]. Polyacrylic acid (PAA), a high molecular weight, water-soluble, biodegradable synthetic polymer made of acrylic acid monomers, is an excellent material for making pH-responsive drug delivery systems [11]. In particular, nanoparticles produced from PAA derivatives can be used to deliver drugs due to their stability, biocompatibility and biodegradability [12]. It is used as a drug carrier due to its low toxicity, antigenicity and immunogenicity [13]. The PAA chains provide not only hydrophilic nature but also a large number of carboxyl groups after being bonded onto the nanoparticles, allowing the drug to be loaded efficiently via electrostatic interactions [12]. Moreover, gelatin is a natural polymer consisting of a large complex polypeptide molecule of amino acids. It is non-toxic, biocompatible, and easily available [14].

Among these, hydrogel-based biodegradable natural materials crosslinked by gamma irradiation offer unique advantages over other drug delivery systems. The gamma irradiation process is a process that is extremely pure, simple to control and known as a “cold, sterilized process” for sensitive materials that cannot be exposed to chemicals or temperature [15]. Furthermore, using gamma irradiation to prepare crosslinked soft hydrogel can provide additional advantages in controlling physical, chemical, and biological properties by tailoring the polymer chain and functionality while accommodating a wider range of monomers with desirable chemistry [16, 17].

Our research group has had a long-standing interest in developing 5- Fluorouracil nanoparticles that can be prepared from biodegradable polymers such as gelatin. Accordingly, this study aimed to synthesize pH-sensitive and biodegradable nanogel-based polyacrylic acid and gelatin via gamma irradiation to overcome the limitations of 5-FUand controlled delivery with a high level to the target tissue as well as evaluate the in vivo antitumor efficacy of 5-FU-loaded Gelatin/PAAc nanogel against DMH-induced colon carcinoma in rats.

## Materials and methods

### Materials

All reagents, monomers of acrylic acid (AAc), gelatin Dimethyl sulfoxide (DMSO), 3-(4,5-dimethylthiazol-2-yl)-2,5-diphenyltetrazolium bromide (MTT) and trypan blue dye were purchased from Sigma (St. Louis, Mo., USA). Fetal Bovine serum, RPMI-1640, HEPES buffer solution, L-glutamine, gentamycin, and 0.25% Trypsin–EDTA were purchased from Lonza (Belgium).

### Irradiation process

Gamma irradiation of polymer/monomer in an aqueous solution was carried out in a ^60^Co Gamma cell instruction at National Center for Radiation Research and Technology (NCRRT), Egyptian Atomic Energy Authority. The irradiation dose was 5KGy and was carried out at a dose of 0.732 kGy/h. On the other hand, Whole-body gamma irradiation was performed using Canadian gamma cell-40 (137Cesium) at a dose rate of 0.67 Gy min − 1 for a total dose of 4 Gy.

### Radiation synthesis of P(G/AAc)/5-FUNanogel

First, the solution of gelatin /5-FU was prepared by dissolving (500 ppm) of 5-fluorouracil and 0.5 g of gelatin in 5 ml of hot water. This gelatin/5-FU solution was poured into 5 ml of ethanol and subjected to stirring at a high-speed homogenizer at 1000 rpm for 45 min. The gelatin/5-FU solution was labeled with an (A) letter. Second, the monomers solution of acrylic acid (AAc) was prepared by dissolving 1.5 ml of acrylic acid in 10 ml of 0.1 M HCl solution. The pH of the homogenized solution of (AAc) monomers was obtained at pH 1. The monomers of (AAc) and gelatin/5-FU solution were mixed and subject to gamma irradiation at a dose of 5 kGy to begin the polymerization reaction. The obtained suspension solution of gelatine-based P(G/AAc)/5-FU was preserved at the temperature of 4 °C.

### Characterization of gelatine-based P(G/AAc)/5-FUnanogel

The particular size and concentration of the prepared nano are important for the biomedical application of the nanoparticles. The morphology of gelatine-based P(G/AAc)/5-FUnanogel was characterized by transmission electron microscopy (TEM, JEOL; model JEM2100, Japan). A sample of gelatine-based P(G/AAc)/5-FU nanogel was analyzed through dynamic light scattering (DLS) and the particle size distribution was recorded on the ZetaSizer Nano ZS particle analyzer (Malvern Instruments Limited). Additionally, both DLS and ZetaSizer were used to evaluate the pH- sensitivity of the gelatine-based P(G/AAc)/5-FU nanogel via soaking in phosphate buffer solution at different pH- values ranging from 1 to 7. Moreover, ultraviolet–visible (UV/Vis) spectroscopy was used to evaluate the release of the 5-FU from gelatine-based P(G/AAc)/5-FU nanogel as well as the chemical stability of the nanogel at 266 nm in response to the pH change.

### Cytotoxicity assay

HCT-116 cells (human colon cancer cell line), were obtained from the American Type Culture Collection (ATCC, Rockville, MD). The cells were grown on RPMI-1640 medium supplemented with 10% inactivated fetal calf serum and 50 µg/ml gentamycin. The cells were maintained at 37 °C in a humidified atmosphere with 5% CO2 and were subcultured two to three times a week.

#### Cytotoxicity evaluation using viability assay

For antitumor assays, the tumor cell lines were suspended in the medium at concentration 5 × 10^4^ cell/well in Corning^®^ 96-well tissue culture plates, then incubated for 24 h. The tested compounds were then added into 96-well plates (three replicates) to achieve ten concentrations for each compound. Six vehicle controls with media or 0.5% DMSO were run for each 96-well plate as a control. After incubating for 24 h, the number of viable cells was determined by the MTT test. Briefly, the media was removed from the 96 well plates and replaced with 100 µl of fresh culture RPMI 1640 medium without phenol red then 10 µl of the 12 mM MTT stock solution (5 mg of MTT in 1 mL of PBS) to each well including the untreated controls. The 96 well plates were then incubated at 37 °C and 5% CO_2_ for 4 h. An 85 µl aliquot of the media was removed from the wells, and 50 µl of DMSO was added to each well and mixed thoroughly with the pipette, and incubated at 37 °C for 10 min. Then, the optical density was measured at 590 nm with the microplate reader(SunRise, TECAN, Inc, USA) to determine the number of viable cells and the percentage of viability was calculated as [(ODt/ODc)] × 100% where ODt is the mean optical density of wells treated with the tested sample and ODc is the mean optical density of untreated cells. The relation between surviving cells and drug concentration is plotted to get the survival curve of each tumor cell line after treatment with the specified compound. The 50% inhibitory concentration (IC_50_), the concentration required to cause toxic effects in 50% of intact cells, was estimated from graphic plots of the dose–response curve for each conc. using GraphPad Prism software (San Diego, CA. USA) [18].

### The LD50 of gelatine-based P(G/AAc)/5-FU nanogel

The LD50 is often an early step in determining and estimating chemical hazardous characteristics. According to the procedure of Bass et al. [19], the LD50 of 5-FU nanogel was estimated in the experimental rats. Doses ranging from 50 to 150 mg/kg b.w. were delivered intraperitoneally to determine the median lethal dose (LD50) of gelatine-based P(G/AAc)/5-FU nanogel. After 24 h, mortality was recorded, and it was calculated as follows:

$${\text{LogLD50}} = {\text{Log\, LD\,next\,below\,50\%}} + ({\text{Log\,increasing\,factor}}\, \times \,{\text{proportionate\,distance}})$$

$${\text{Proportionate \,distance}}=\frac{50\%-{\text{Mortality \,next \,below 50\%}}}{\text{\% mortality\, above \,50\%}}-{\text{mortality \,next \,below\,50\%}}$$

### Animals

The care and use of laboratory animals were carried out according to the protocols approved by the Institutional Animal Ethical Committee in accordance with the international guidelines for animal experimentation. Additionally, the present study was approved by the Institutional Animal Care and Use Committee Research Ethic Board (BUFVTM 04-04-22). Male rats (100–120 g) were obtained from the Institutional animal house and placed in clean cages at 22 ± 2 °C, and a constant 12 h light/ dark cycle with free access to a commercial pellet diet and drinking water.

#### Experimental design

Using 1,2-dimethylhydrazine (DMH), a well-established colon carcinogen that was metabolized in the liver into azoxymethane, a colon cancer model was created in the current study in rats. As a result, the screening of antitumor compounds was made possible by using DMH to induce colon tumors in rats, a model that resembles human diseases [20]. 1,2-dimethylhydrazine (DMH) was diluted with sterile saline. Rats received weekly doses of DMH (20 mg/kg) by subcutaneous (s.c.) injections once a week for 8 weeks [21], then exposed to a single dose of gamma radiation (IR) (4 Gy) to promote the carcinogenesis of the colon. At the scheduled time, surviving rats were anesthetized with urethane and then sacrificed by cervical dislocation. Colon tissues were excised and washed immediately with ice-cold saline for further analysis.

#### Experimental Groups: rats were distributed to four experimental groups (10 rats in each group).

- *Group 1* Normal rats served as control.
- *Group 2* Rats injected with DMH (s.c) and exposed to IR to induce colon dysplasia.
- *Group 3* DMH + 5-FU treated group where rats received 5-FU (12.5 mg/kg b.wt) three times weekly by intraperitoneal injection [22].
- *Groups 4* DMH + P(G/AAc)/5-FU treated group where rats received P(G/AAc)/5-FU (2.5 mg/kg b.wt) three times weekly by intraperitoneal injection.

#### Biochemical measurements

The colon homogenates were used for estimating the level of microtubule-associated protein light chain 3B (LC3-II) (Cat.No MBS1600540), Nuclear Factor Kappa B (NFkB) (cat. no. MBS453975) and Toll-Like Receptor 2 (TLR2) (cat.no. MBS2022789) by a rat ELISA kit obtained from MyBioSource, Inc. San Diego, USA. The absorbance at 450.0 nm was determined by using a microplate reader (Bio-Rad model 680, USA).

#### Real-time quantitative polymerase chain reaction determination

*RNA extraction and cDNA synthesis* To investigate the changes in mRNA expression for PI3K, Becline 1, ATG7, Akt1, mTOR, P62*,* Caspase 9, and Caspase3 genes, total RNA was isolated from 50 mg liver tissue using TRIzol reagent (Invitrogen) according to Chomczynski [23]. First-strand complementary DNA (cDNA) synthesis was performed using reverse transcriptase (Invitrogen) using template 1 mg RNA.

##### Quantitative real-time polymerase chain reaction (qPCR)

RT-PCRs were performed in a thermal cycler step one plus (Applied Biosystems, USA) using Sequence Detection Software (PE Biosystems, CA). The oligonucleotides utilized in these experiments are listed in Table 1. A reaction mixture of a total volume of 25 ml consisting of 2 SYBR Green PCR Master Mix (Applied Biosystems), 900 nM of each primer and 2 ml of cDNA. PCR thermal-cycling conditions included an initial step at 95 °C for 5 min; 40 cycles at 95 °C for the 20 s, 60 °C for 30 s, and 72 °C for 20 s. Relative expression of Phosphoinositide 3-kinases (PI3Ks), Becline 1, Autophagy related 7 (ATG7), Protein kinase B (Akt), mammalian target of rapamycin (mTOR), P62, Caspase 9 and Caspase3 genes mRNA were calculated using the comparative Ct method according to Pfaffl [24] Calculations were performed by calculating the values of the D cycle threshold (DCt) by normalizing the average Ct value of each treatment compared to the endogenous control β-actin. The primer sequences are shown in Table 1.

**Table 1:** Primer sequences used for RT-PCR

Primer	Sequence
PI3K	Forward:5ʹ—AACACAGAAGACCAATACTC—ʹ3 Reverse:5ʹ—TTCGCCATCTACCACTAC—ʹ3
Becline 1	Forward:5ʹ—CGGAATTCTATGGAAGGGTCTAAGACGTCC—3ʹ Reverse:5ʹ—CGGGATCCTCATTTGTTATAAAATTGTGAGGACA—3ʹ
ATG7	Forward 5ʹ—GCTGGTCTCCTTGCTCAAAC—3ʹ Reverse:5ʹ —CAGGGTGCTGGGTTAGGTTA—3ʹ
Akt1	Forward:5ʹ—GTGGCAAGATGTGTATGAG—3ʹ Reverse: 5ʹ—CTGGCTGAGTAGGAGAAC—3ʹ
mTOR	Forward:5ʹ—GGTGGACGAGCTCTTTGTCA—3ʹ Reverse: 5ʹ —AGGAGCCCTAACACTCGGAT—3ʹ
P62	Forward:5ʹ—TCCTGCAGACCAAGAACTATGACATCG—3ʹ Reverse: 5ʹ—TCTACGCAAGCTTAACACAACTATGAGACA—3
Caspase 9	Forward:5ʹ—AGCCAGATGCTGTCCCATAC—3ʹ Reverse: 5ʹ—CAGGAGACAAAACCTGGGAA—ʹ3
Caspase3	Forward:5ʹ—GGTATTGAGACAGACAGTGG—3ʹ Reverse: 5ʹ—CATGGGATCTGTTTCTTTGC—ʹ3
β-actin	Forward: 5ʹ —AAGTCCCTCACCCTCCCAAAAG—ʹ3 Reverse: 5ʹ—AAGCAATGCTGTCACCTTCCC—3ʹ

#### Immunoblotting analysis

The colonic tissue proteins have been removed by TRIzol and a protein content measurement has been taken using the Bradford technique. Protein samples were subjected to 10% SDS-PAGE and then transferred onto PVDF membranes. The membranes were blocked with 5% skim milk for 60 min at room temperature. Then, they were incubated with primary antibodies to total and phosphorylation of both 5ʹ AMP-activated protein kinase (AMPK), BCL2 Associated X, Apoptosis Regulator (BAX), B-cell lymphoma 2 (BCL2) and polyclonal antibody anti- β -actin rabbit as a control (Santa Cruz) overnight at 4 °C. Next, they were washed with phosphate-buffered saline (PBS) and tween 3 to 4 times, for 120 min, incubated with secondary monoclonal antibodies coupled with horseradish peroxidase. The membranes were subsequently washed four times with PBS at room temperature. With the Amersham detection kit, chemiluminescence was used to visualize membranes according to the manufacturer’s instructions and thereafter subjected to X-ray film. Cell Signaling Technologies, USA have been used to acquire primary and secondary antibodies [25].

#### Histological examination

Colon tissue specimens were fixed in 10% neutral buffered formalin. The fixed specimens were then trimmed, washed and dehydrated in ascending grades of alcohol, cleared in xylene, embedded in paraffin, sectioned at 4–6 μm thickness and stained with hematoxylin and eosin used conferring to Bancroft and Stevens. The colon sections were viewed on a light microscope (Olympus BX 41, Japan) and photographed using Olympus digital camera [26].

#### Immunohistochemical analysis

Paraffin-embedded tissue sections were deparaffinized and rehydrated with graded ethanol dilutions, after which antigen retrieval was carried out. Caspase-3 (Santa Cruz Biotechnology, USA), an apoptotic marker and Ki67 (Santa Cruz Biotechnology, USA), a proliferative marker, in the colon were assessed histochemically [27]. The immunohistochemical staining for Caspase-3 and Ki67 was semi-quantitively assessed in high microscopic power fields (40X) as described by Gerdes et al. [28]. Two main criteria, the color intensity and the percentage (%) of positively stained cells were used for this assessment. For color intensity, a grading system scaled from 0 to 3 was used, in which grade 0 = no staining, graded 1 = weak staining, graded 2 = moderate staining and graded 3 = strong staining. Similarly, a grading system scaled from 0 to 3 was used for assessing the percentage (%) of positively stained cells in high power fields, in which grade 0 denotes 0%, grade 1denotes < 30%, grade 2 denotes 30–70% and grade3 denotes > 70%. The total immunoreactivity score (IRS) of each stained section is the sum of these two criteria.

### Statistical analysis

Differences in the mean of variables were calculated among all experimental groups using a one-way analysis of variance (ANOVA). The results have been expressed as mean ± standard deviation (SD). For all test results, SPSS statistical software version 20 for Windows (SPSS^®^ Chicago, IL, USA) has been used. Variations for all analyses were statistically significant at p < 0.05. The charts were graphed via GraphPad Prism 8 (GraphPad, CA, USA).

## Results

### Characterization of nanoparticles

**Fig. 1:**
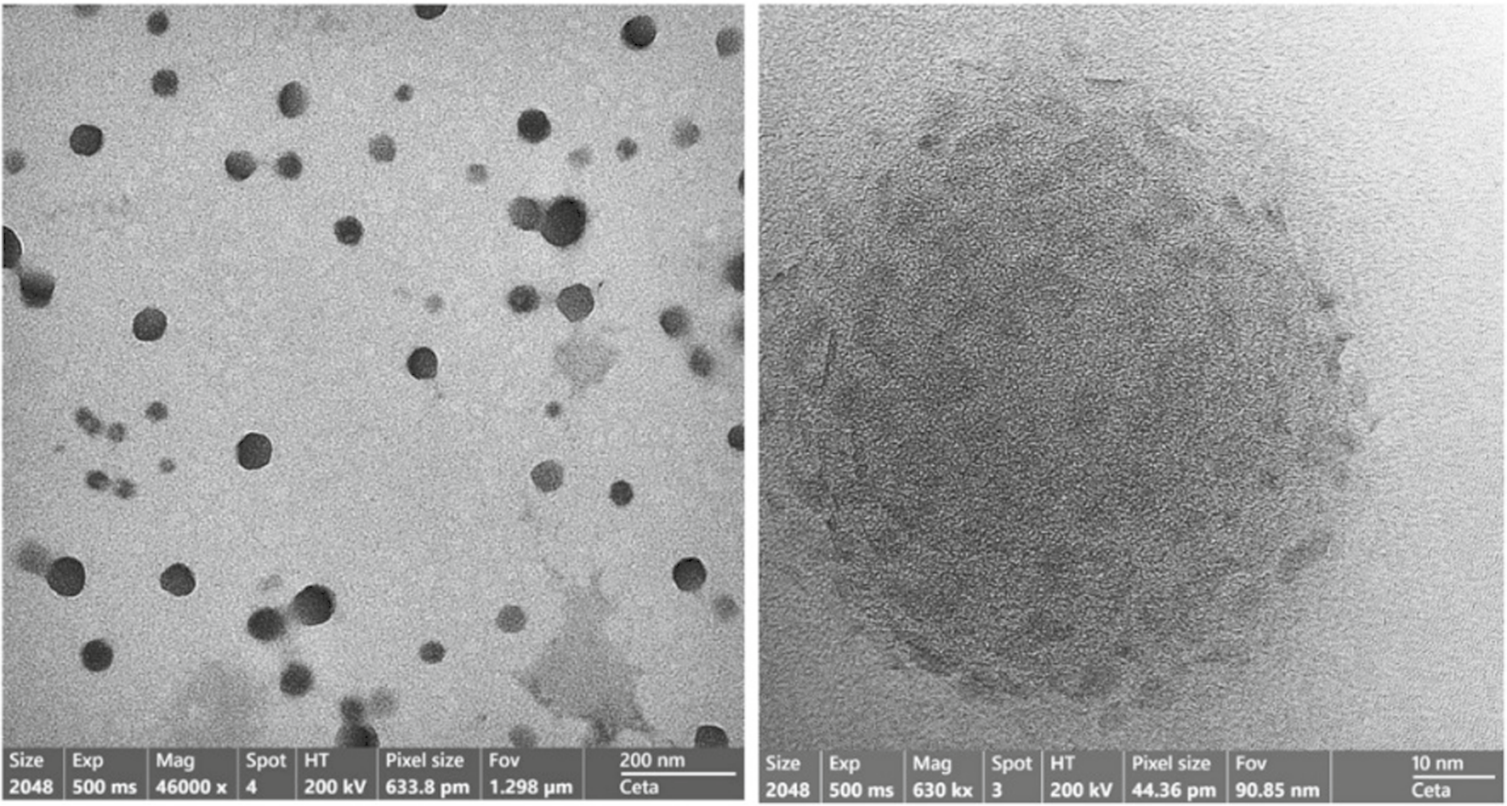
TEM analysis of the gelatine-based P(G/AAc)/5-FU nanogel

**Fig. 2:**
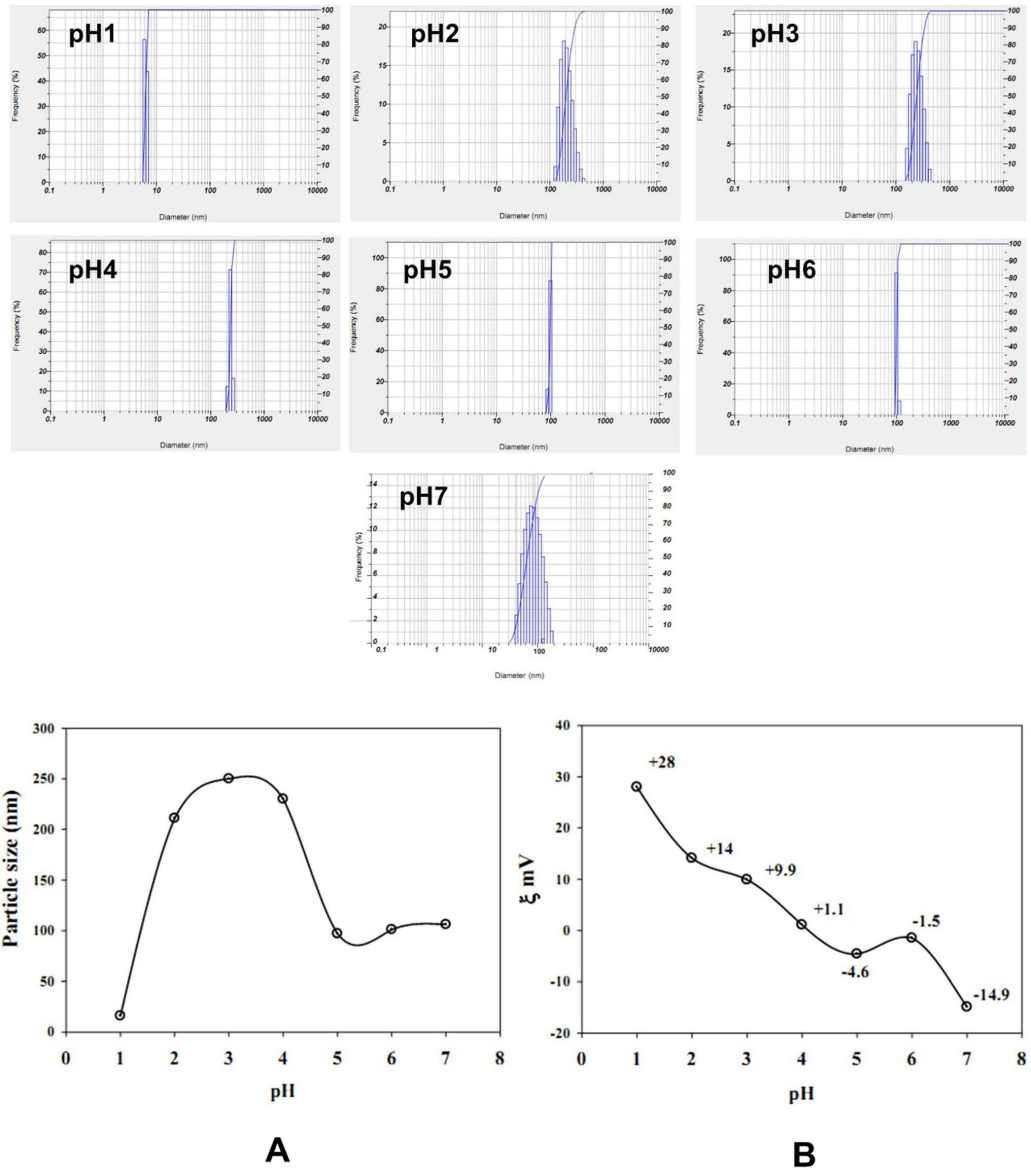
The effect of different pH on the particle size **A** and Zeta potential **B** of gelatine-based P(G/AAc)/5-FU nanogel

Using biodegradable nanoparticles such as nanogels with tiny sizes for drug delivery ameliorates the in vivo stability and the incorporation of the drug as well as its delivery leading to excellent therapeutic efficiency. The properties of gelatine-based P(G/AAc)/5-FU nanogel were characterized by TEM, DLS, and UV/Vis spectrophotometry. The TEM analysis showed that the prepared gelatine-based P(G/AAc)/5-FU nanogel appeared as a dark dot spherical shape with good monodispersity (Fig. 1). The DLS analysis shown in Fig. 2A confirmed that all gelatine-based P(G/AAc)/5-FU complexes samples exhibited particle size on the nanoscale ranging from 16 to 106 nm. The smallest particle size (16 nm) of gelatine-based P(G/AAc)/5-FU nanogel was obtained at pH 1. However, a marked increase in the particle size was observed when pH moved up from 2 to 4. Meanwhile, increasing the pH values from 4 to 7 resulted in a significant decrease in the particle size. Additionally, Fig. 2B showed that the Zeta potentials of the gelatine-based P(G/AAc)/5-FU nanogel samples were between + 28 mV at pH1 and − 14.9 mV at pH7.

**Fig. 3:**
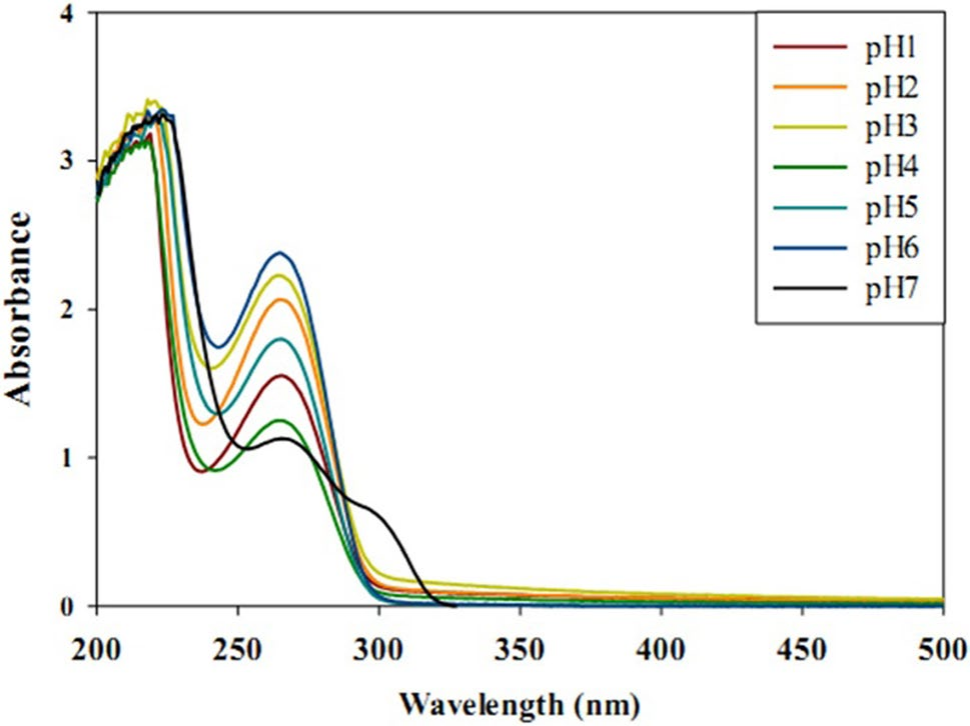
The UV/Vis spectrophotometers of the drug released at different pHs from gelatine-based P(G/AAc)/5-FU nanogel

Furthermore, the results obtained from the UV/Vis spectrophotometry showed a stable peak (Fig. 3) that is nearly similar to each other confirming the higher stability of the gelatine-based P(G/AAc)/5-FU nanogel even at different pH. Moreover, the release of 5-FU from the gelatine-based P(G/AAc)/5-FUnanogel was almost higher at pH 1, 3 and 6 than at pH 7 while the lowest release of the 5-FU was seen at pH 4. The pH-dependent release of the P(G/AAc)/5-FU complex samples is controlled by the degree of dissociation of PAAc molecules. This may be due to the sensitivity of the carboxylic groups (–COO^−^) of acrylic acid towards pH change. At pH 6 the negatively charged (–COO^−^) increase the electrostatic repulsion force within the crosslinked polymer networks leading to the diffusion of water molecules in the inner structure of the hydrogels causing the swelling degradation of the nanogel and consequently the release of the loaded 5-FU. It was reported that the pH value was 6 in tumor cells due to the anaerobic glucose metabolism. Based on this, the gelatine-based P(G/AAc)/5-FU nanogel may expect to potentiate the antitumor efficiency of the 5-FU depending on the targeted delivery and release inside the tumor tissue.

### The inhibitory concentration (IC50)

**Fig. 4:**
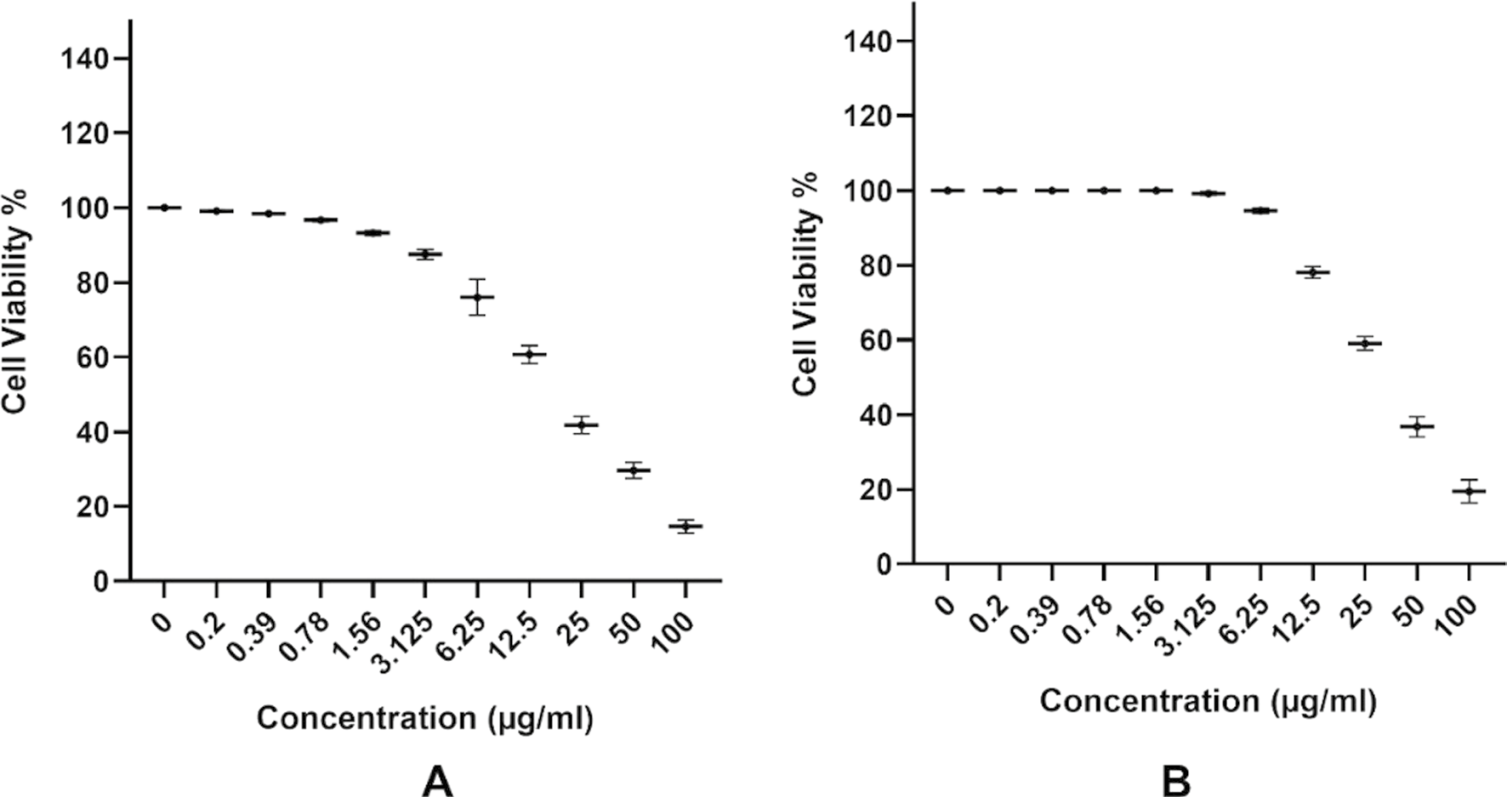
Inhibitory effect of 5-FU**A** and 5-FU nano gel **B** on cell viability of HCT-116 cell line

The 50% growth inhibitory concentration (IC50) values were calculated as the concentration of the compound that inhibited the viability of cells by 50% as compared with control cells grown in the absence of an inhibitor. It was found that both 5-FU and P(G/AAc)/5-FU nanogel used concentrations from 0.00 μg/ml to 100 μg/ml dramatically inhibited the growth of the HCT-116 cell lines in a dose-dependent manner with calculated IC50 values of 19.64 ± 1.78 µg/ml and = 35.22 ± 3.04 µg/ml respectively (Fig. 4).

### Results of LD50 of gelatine-based P(G/AAc)/5-FU nanogel

From the results of LD50 represented in Table (2), the concentration of the prepared gelatine-based P(G/AAc)/5-FU nanogel was found to be 124.5 mg/kg body and the tenth of this dose weight was used for the intraperitoneal injection. This dose revealed that the gelatine-based P(G/AAc)/5-FU nanogel was safe and did not cause any side effects.

**Table 2:** Median lethal dose of

Dose (mg/Kg b.wt.)	Number of animals	survivals	Mortality	Deaths %
150	10	0	10	100
140	10	2	8	80
130	10	5	5	50
120	10	7	3	30
100	10	9	1	10
50	10	10	0	0

$${\text{LogLD50}} = {\text{LogLD\,next\,below\,50\%}}\,+ \left( {{\text{Log\,factor}}} , \times \,{\text{proportionate\,distance}} \right).$$

$$\mathrm{Increasing \,factor }(\mathrm{approx}.)=1.077$$

$$\mathrm{Proportionate\, distance}=0.5$$

$$\mathrm{Log }{\mathrm{LD}}_{50}=\mathrm{Log }\left(130\right)+(\mathrm{Log }1.077\times 0.5).$$

$$\mathrm{LD}50=124.5\mathrm{ mg}/\mathrm{Kg \,body \,weight}.$$

### Histopathological examination

**Fig. 5:**
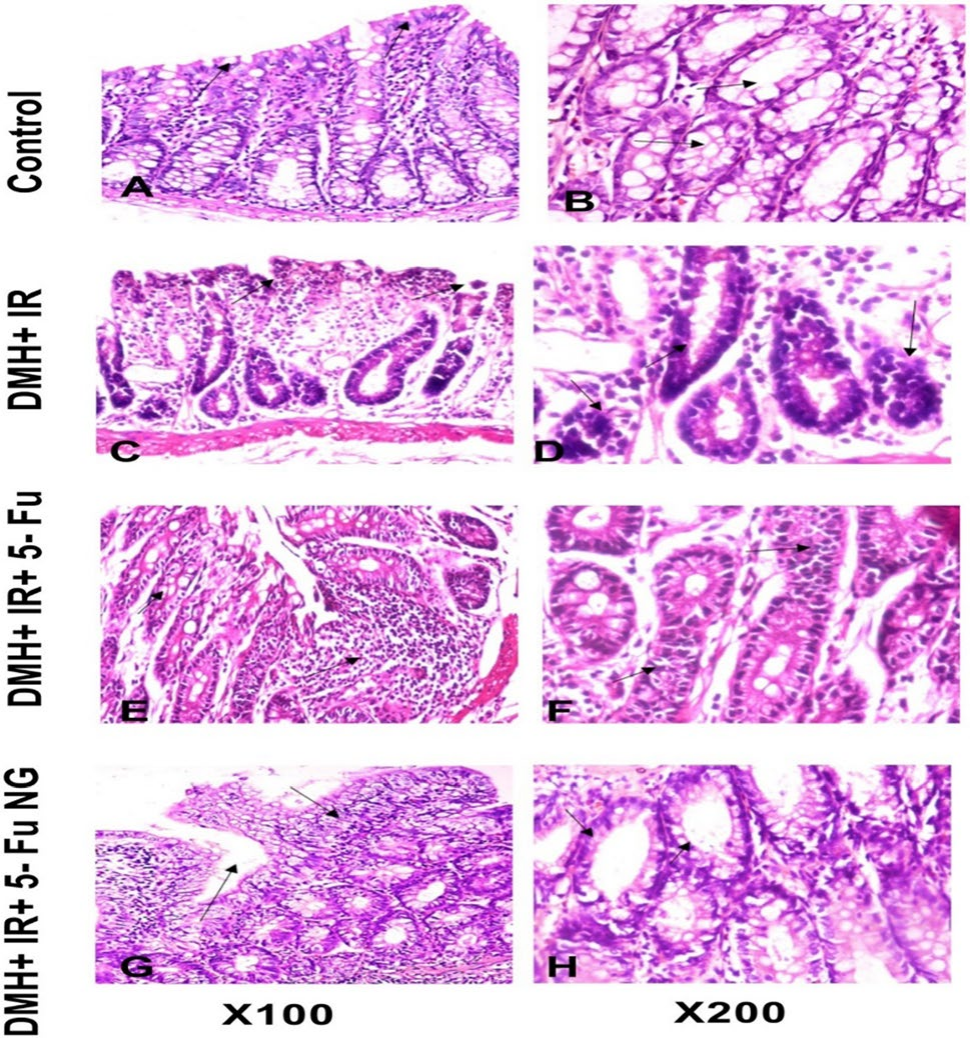
Photomicrograph of the colon tissue section. Figures (**A** and **B**) representing control showed a normal histological structure of the colon. However, in Figure (**C** and **D**) the DMH + IR showed hyperplasia of the mucosal glands, dysplastic changes, and inflammatory cell infiltration. Figure (**E** and **F**) colon tissues after treatment with 5-FU showed moderate dysplasia and some inflammatory cells. Furthermore, 5-FU nanogel lowered the hyperplasia and the inflammatory cells (figure **G** and **H**)

The photomicroscopic image of the control group section exhibited normal histological structure of the colon tissue consisting of mucosa, submucosa, muscularis, and serosa/adventitia. The mucosa is composed of villi (lined by a single layer of columnar cells with oval basal nuclei), goblet cells scattered in between, and intestinal crypts in addition to the intestinal glands (Fig. 5A–B). However, the injection of the DMH and exposure to IR showed dysplastic changes such as aberrant crypt foci (ACF) with narrow lumens in epithelial cells and intestinal glands with irregular shape and size, loss of goblet cells polarity, increased mitotic activity as well as the presence of complex glands with some areas of solid growth grade (4). Moreover, subepithelial edema and inflammatory cell infiltration mainly lymphocytes and macrophages were noticed in lamina propria and submucosa score (3) (Fig. 5C–D). On contrary, treatment with 5-FU showed a moderate degree of cell dysplasia with irregular shape and size with some complexity of glandular structure grade (2) and damaged crypts with sloughing epithelial cells. Additionally, a small number of goblet cells with some inflammatory cells score (2) (Fig. 5E–F) were detected. While treatment with 5-FU nanogel lowered the hyperplasia of the mucosal glands and ameliorated the dysplastic changes, goblet cells and damaged crypts grade (1). Furthermore, it reduced the inflammatory cell infiltration in the lamina propria of the mucosa, and submucosa score (1) (Fig. 5G–H).

### Effect of 5-FU and 5-FU nano gel on TLR2 and NF-κB levels in colon tissues

**Fig. 6:**
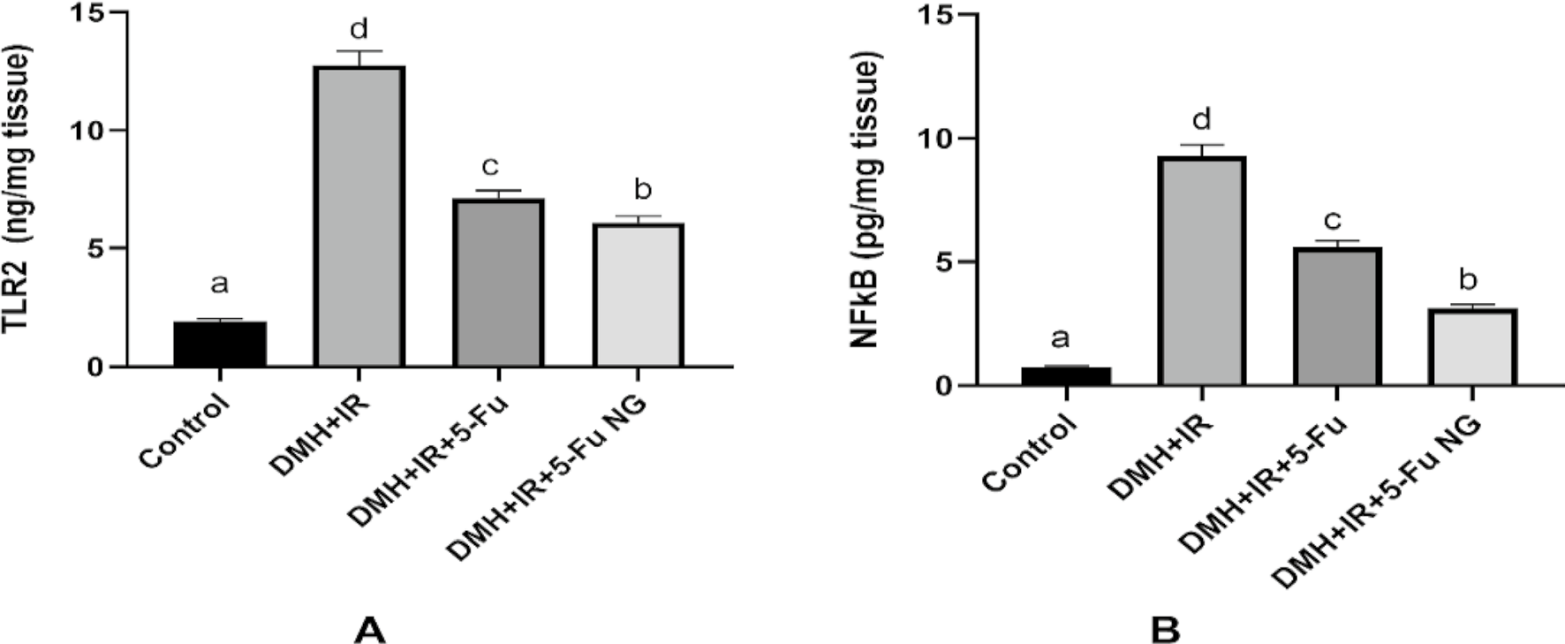
Effect of 5-FU and 5-FU nanogel on TLR2 **A** and NF-κB **B** levels in colon tissues. Values were expressed as Means ± SD ( n = 6). Values sharing different letters are significantly different (P < 0.05) while those with similar letters are non-significant

Toll-like receptors (TLRs) have a potential role in immunity both innate and adaptive. Abnormal activation of TLR promotes inflammation and carcinogenesis. Herein, as shown in Fig. (6), a notable increase in the levels of TLR2 was observed in the rats injected with DMH and exposed to γ-IR to induce colon cancer supporting its role in colorectal pathogenesis. In contrast, a significant decline was noticed after treatment with 5-FU or 5-FU nanogel. It was reported that the heterodimerization of TLR2 triggers the activation of PI3K/Akt and NF-κ B signaling pathways which were involved in cellular proliferation. Consequently, the higher levels or the activation of the TLR2 was accompanied by increased levels of the NF-κB in the colon tissues of the rats injected with DMH + IR compared to the control. The activation of NF-κB signaling contributes to proliferation, cell survival and carcinogenesis.

### Effect of 5-FU and 5-FU nanogel on the PI3K/ AKT/ mTOR signaling pathway

**Fig. 7:**
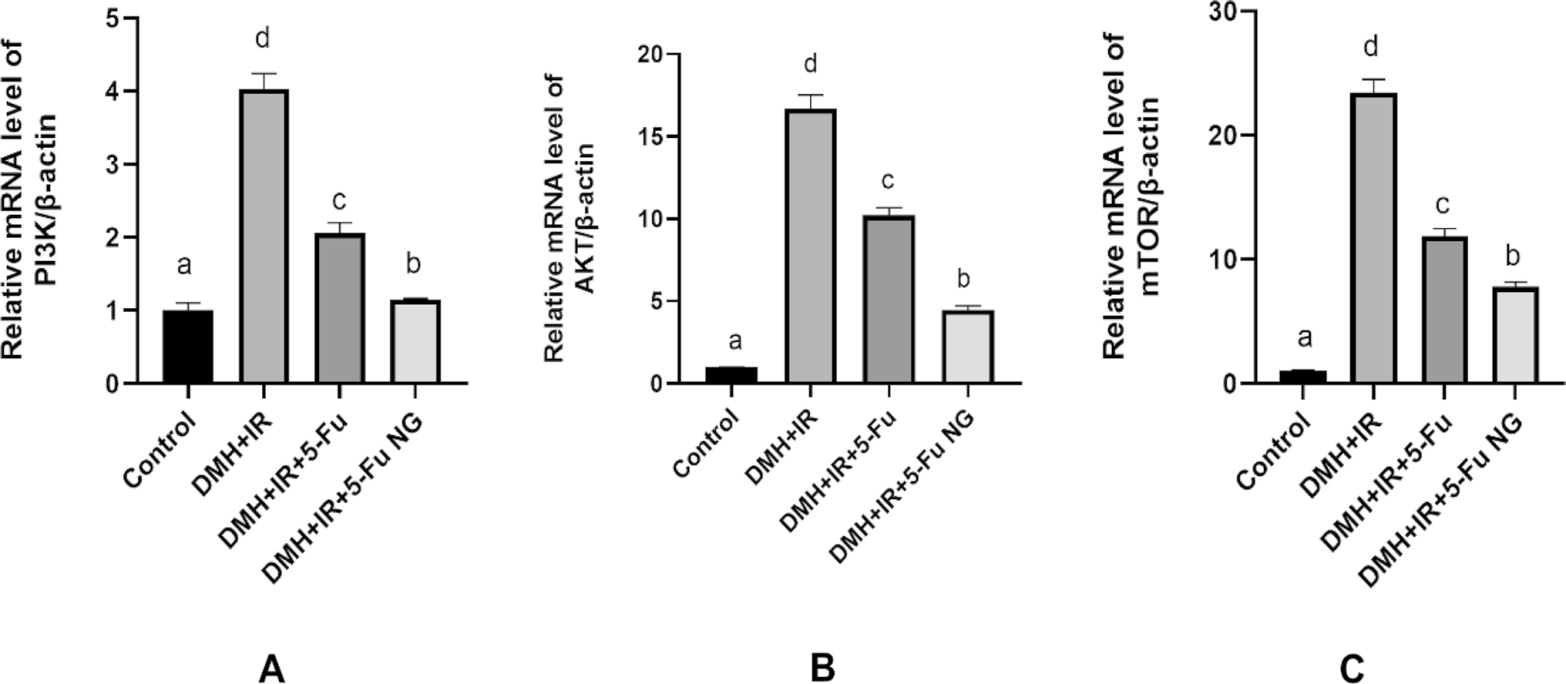
Effect of 5-FU and 5-FU nanogel on the gene expression of PI3K **A**, AKT **B**, and mTOR **C** in colon tissues. Values were expressed as Means ± SD ( n = 6). Values sharing different letters are significantly different (P < 0.05) while those with similar letters are non-significant

Based on the important role of the PI3K/Akt/mTOR signaling pathway in cell metabolism, survival, proliferation, and tumor progression, the gene expressions of PI3K, AKT, and mTOR were determined by RT-PCR in the colon tissues. The obtained results revealed that the injection of the DMH and exposure to γ-IR remarkably upregulated the mRNA levels of the PI3K, AKT, and mTOR relative to the control indicating the high proliferation activity of the cancerous cell in the colon. However, upon treatment with 5-FU or 5-FU nanogel there was an effective reduction in the gene expression of the PI3K, Akt and mTOR confirming the inhibitory potential against the hyperactivated PI3K/Akt/mTOR signaling pathway in colon cancer (Fig. 7). Moreover, the amplification curves representing the Ct values, the raw data and the calculation of the ΔΔCt value of PI3K, AKT, and mTOR genes were shown in Figure S1, Figure S2, Figure S3, Supplementary Table 1, Supplementary Table 2 and Supplementary Table 3 respectively in the supplementary file.

### Effect of 5-FU and 5-FU nanogel on the proliferation

**Fig. 8:**
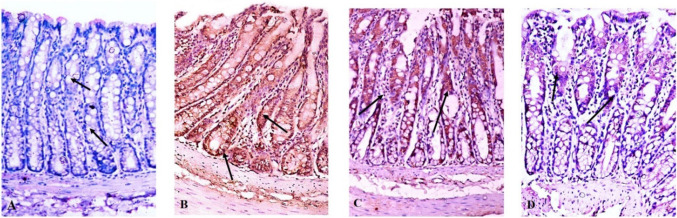
Effect of 5-FU and 5-FU nano gel on the expression of the proliferation marker Ki-67 by IHC. Figure **A** control group, **B** DMH + IR group, **C**: DMH + IR + 5-FUgroup and **D**: DMH_IR + 5-FUnanogel

Additionally, another specific marker for cell proliferation the expression of Ki-67 protein was detected by Immunohistochemistry. the tissues with colon cancer induced by DMH + IR showed overexpression of the Ki-67 marker represented by the presence of more than 70% of the strongly positive stained cells (grade 3) compared to the control group which did not show any significant Ki-67 expression, assuring a higher proliferation level in the rat's colon tissues. On contrary, the higher expression of the Ki-67 was declined upon treatment with 5-FU showing a moderate number of positive cells (grade 2 denotes 30–70%) in comparison with the DMH + IR group (figure). Moreover, the 5-FU nanogel downregulated the expression and percentage of the Ki-67 positive stained cells with weak cytoplasmic and/or nuclear staining to less than 30% (grade 1) as shown in Fig. (8).

### Effect of 5-FU and 5-FU nanogel on the autophagy

**Fig. 9:**
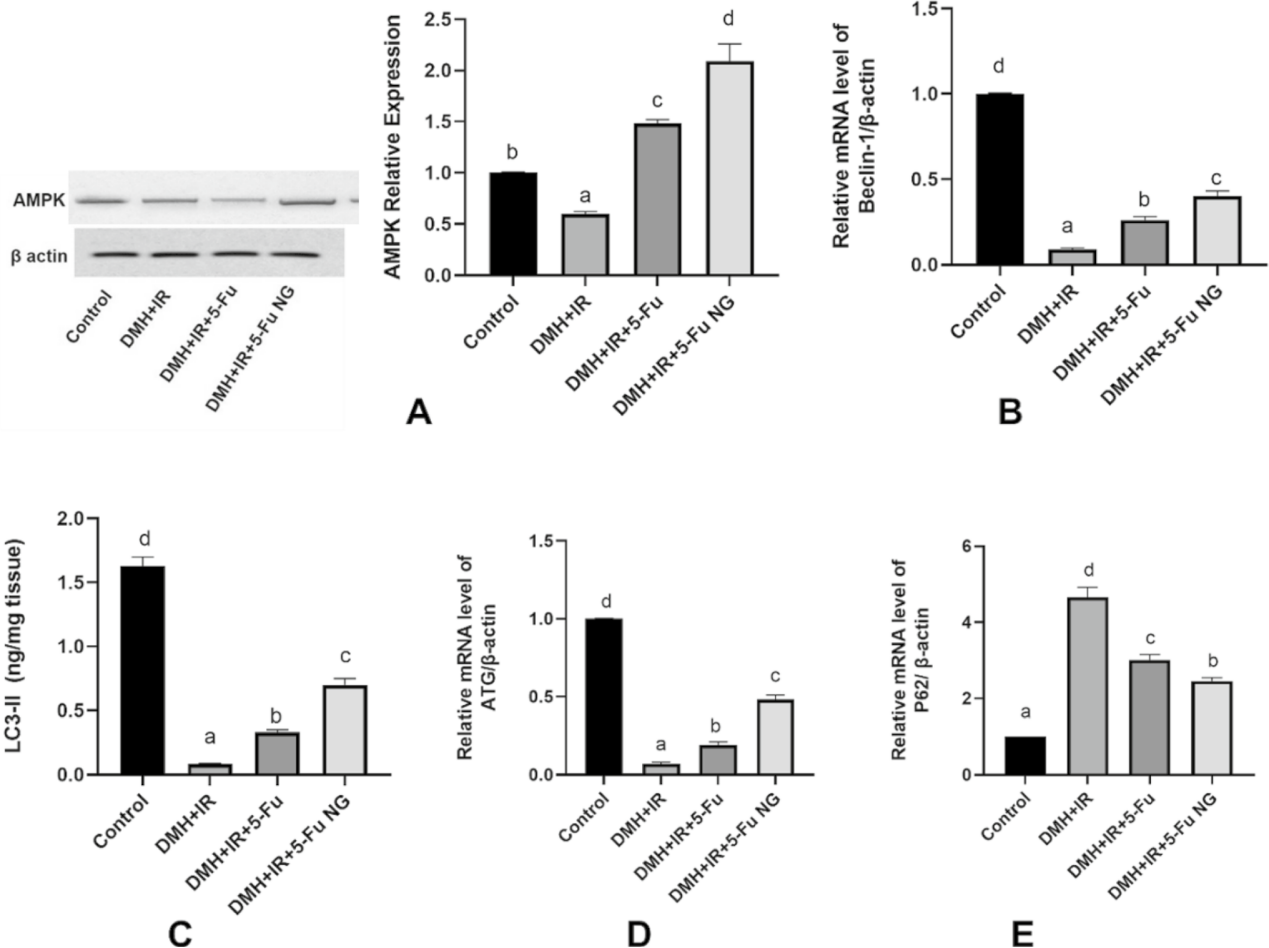
Effect of 5-FU and 5-FU nanogel on autophagy. Western blot analysis of AMPK **A**, the gene expression of Beclin-1 **B**, levels of LC3-II **C** and the gene expression of ATG7 **D**, and P62 **E** in colon tissues. Values were expressed as Means ± SD (n = 6). Values sharing different letters are significantly different (P < 0.05) while those with similar letters are non-significant

Carcinogenesis and proliferation are enhanced with activation of the PI3K/AKT/mTOR pathway concomitant with inhibition and dysregulation of autophagy. Autophagy is a normal multi-step process of self-degradation of the intracellular components and organelles by lysosomes to maintain cellular homeostasis under stress. It is mainly regulated by both PI3K/Akt/mTOR and AMPK/mTOR signaling pathways besides, other related autophagy signaling pathways. Therefore the levels of regulator signals of autophagy were analyzed by RT-PCR, ELISA and western blot. The current data in Fig. (9) revealed that the overexpression of the mTOR in the colon tissues was associated with the suppression of the regulator of autophagy including the protein levels of AMPK, mRNA of the Beclin1 and ATG7 as well as the level of the LC3-II coupled with the upregulated P62 gene expression in the DMH + IR group relative to the control group. The dysregulated autophagy and accumulated P62 promote oncogenesis. Conversely, treatment either with 5-FU or 5-FU nanogel inhibited the PI3K/ AKT/mTOR which consequently, activated the AMPK/mTOR pathway by increasing the levels of AMPK protein and LC3-II in addition to the gene expression of the Beclin1 and ATG7 along with lowering the mRNA of the P62. Thus activating and initiating autophagy. Moreover, the amplification curves representing the Ct values, the raw data and the calculation of the ΔΔCt value of Beclin1, ATG7, and P62 genes were shown in Figure S4, Figure S5, Figure S6, Supplementary Table 4, Supplementary Table 5 and Supplementary Table 6 respectively in the supplementary file. The Quantitative western blotting of AMPK protein was shown in Figure S9 in the supplementary file.

### Effect of 5-FU and 5-FU nanogel on apoptotic markers in colon tissue

**Fig. 10:**
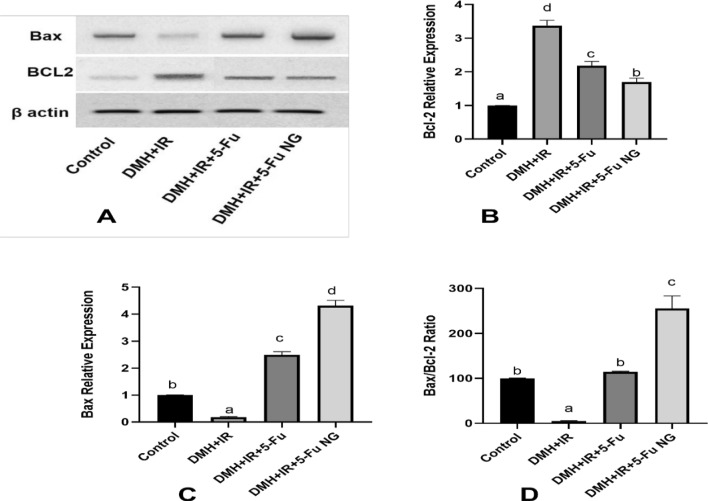
Effect of 5-FU and 5-FU nanogel on mitochondrial apoptosis. Western blot analysis of Bax, Bcl-2 and β-actin protein **A**, Quantitative western blotting analysis of Bcl-2 **B** and Bax **C**, Bax/ Bcl-2 ratio **D** in colon tissues. Values were expressed as Means ± SD (n = 6). Values sharing different letters are significantly different (P < 0.05) while those with similar letters are non-significant

Apoptosis is the physiological process of a programmed cell death that is abolished in cancer cells due to the higher proliferation activity triggered by the aberrant activation of the PI3K/Akt/mTOR pathway together with the dysregulated autophagy which is considered the upstream control of apoptosis death. Accordingly, the expression of the Bcl-2 family proteins such as antiapoptotic Bcl-2 and the pro-apoptotic Bax proteins as well as the mRNA expression of the caspase cascades (3 and 9) were investigated by western blot and RT-PCR respectively in addition to the protein expression of Caspase-3 by immunohistochemistry. The obtained data shown in Fig. (10) revealed a dramatic overexpression of the anti-apoptotic protein Bcl-2 coupled with marked downregulation of the pro-apoptotic protein Bax in the colon tissues of the rats in the DMH + IR group as compared to the control group confirming the impaired mitochondrial apoptosis. Furthermore, the Bax/ Bcl-2 ratio indicates the sensitivity of cells to apoptosis was lower in the DMH + IR group thus favoring tumor growth. On the other hand, the expression levels of the Bcl-2 were downregulated whereas that of the Bax were upregulated after treatment with 5-FU or 5-FU nanogel. Moreover, the Bax/ Bcl-2 ratio increased remarkably hence, sensitizing colon cancer cells toward apoptosis. Moreover, the amplification curves representing the Ct values, the raw data and the calculation of the ΔΔCt value of caspase9, and caspase3 genes were shown in Figure S7, Figure S8, supplementary Table 7 and Supplementary Table 8 respectively in the supplementary file. The Quantitative western blotting of Bax and Bcl2 proteins were shown in Figure S10 and Figure S11 respectively against β- actin (figure S12) in the supplementary file.

**Fig. 11:**
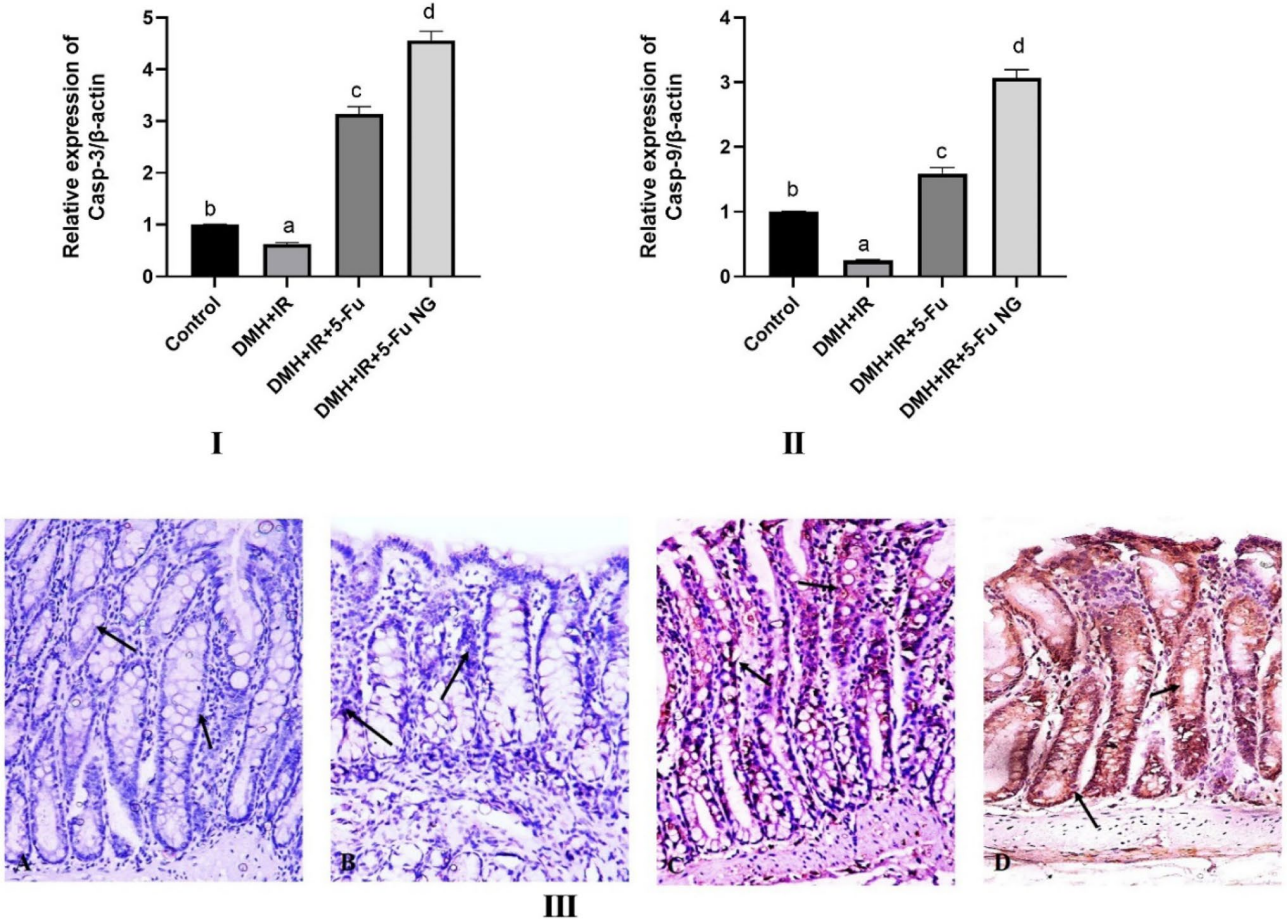
Effect of 5-FU and 5-FU nanogel on caspase cascade. Figures **I** and **II** display the gene expression of caspase 3 and 9 respectively. While figure **III** exhibited the IHC of the caspase 3 protein. Values were expressed as Means ± SD (n = 6). Values sharing different letters are significantly different (P < 0.05) while those with similar letters are non-significant

Additionally, the results displayed a remarkably suppressed activity of both caspase -3 and 9 demonstrated by a diminished mRNA expression in the DMH + IR group relative to the control group. However, treatment with 5-FU or 5-FU nanogel enhanced the activity of both caspase − 3 and 9 by increasing their gene expression (Fig. 11, I and II). Furthermore, the IHC results showed the absence of caspase-3 expression in the colon tissues of the control group (grade 0) (Fig. 11IIIA). While weak staining of the caspase-3 expression is less than 30% (grade 1) in the colon of the DMH + IR group (Fig. 11IIIB). Conversely, treatment of the DMH + IR with 5-FU displayed moderate positive staining of caspase-3 (30–70%, grade 2) (Fig. 11IIIC). Additionally, colon cancer tissues treated with 5-FU nanogel demonstrated high expression of caspase-3 more than 70% with strong cytoplasmic and/or nuclear staining (grade 3) which indicates induction of apoptosis and proves the efficiency of the 5-FU nanogel over the 5-FU(Fig. 11IIID).

## Discussion

Using biodegradable nanoparticles such as nanogels (three-dimensional crosslinked polymer networks that undergo swelling instead of dissolution in an aqueous environment) with a tunable size for drug delivery boosts the in vivo stability and the incorporation of the drug as well as its delivery leading to excellent therapeutic efficiency [29]. Accordingly, our study was designed to prepare 5-FU nanogel as a new form of the colon cancer chemotherapeutic drug 5-FU to avoid the drawbacks of using its pure form including the toxicity of many normal tissues and various side effects [30]. In addition to improving its lower therapeutic effects associated with its poor pharmacokinetics and short half-life [31]. Regarding the outstanding roles of nano-based drug delivery, we suggest that the conjugation of nano-carriers with PI3K/Akt/mTOR inhibitors could be a promising strategy to protect the drugs from degradation, adjust the physicochemical features of drugs, augment the efficacy through high local concentration of cargoes, and reduce the off-target toxicities as this method was utilized with other treatment agents [32].

Normally, the intracellular PI3K/AKT signaling pathway has been involved in regulating cellular growth, differentiation, proliferation, growth, invasion, and metabolism [33]. However, its overexpression or dysregulation exhibited its oncogenic role in various types of cancer, particularly colon cancer where it was implicated in the initiation, development and progression events (proliferation, metastasis, survival, and angiogenesis as well as inhibition of apoptosis) [34, 35]. Previous studies reported the cross-talk between the PI3K/Akt/mTOR pathway with different signaling pathways such as TLR/ NF-κB and AMPK/mTOR in addition to the apoptotic pathways. Accordingly, targeting this pathway is a prominent strategy for colon cancer therapy [36].

In this respect, our results revealed a notable increase in the levels of both TLR2 and NF- κB in the colon tissues of rats injected with DMH and exposed to γ radiation. Proenca et al. [37] reported that TLR2 is one of the TLRs that is remarkably increased in colorectal cancer where it promotes tumor cell proliferation and can be used as a marker for colorectal cancer in humans. Liu et al. [38] revealed that higher levels of TLR2 favor colorectal tumorigenesis and cell growth via its heterodimerization which subsequently activated the PI3K/Akt, NF- κB pathways along with upregulation of various anti-apoptotic genes such as Bcl-2. In contrast, it was indicated that inhibiting or knocking out TLR2, was associated with marked suppression of colon cancer cell proliferation [39].

The previous results of Terzic et al. [40] depicted the abnormal activation of the NF-κB in more than 50% percent of colorectal tumors. Moreover, Soleimani et al. [41] reported that activated NF-κB cascade potentiated colon cancer proliferation, survival, angiogenesis, and metastasis leading to its progression via upregulation of the anti-apoptotic Bcl-2 expression, hence inhibiting the cellular apoptosis [42]. However, suppressing or blocking the NF-κB signaling delayed the CRC progression through the induction of apoptosis in colon cancer cells [41, 43].

NF-κB signaling is not only activated by TLRs but also can be activated in different ways such as DNA damage, stress, and upstream kinases, particularly the oncogenic PI3K/AKT signaling pathway [44, 45]. Activated AKT triggers phosphorylation of IκB kinase IKK-α, leading to IκB degradation and nuclear translocation of NF-κB, therefore, activation of NFκB [46]. Additionally, Kumar and Agnihotri [47] observed that induction of colon cancer by DMH + DSS was associated with a significant rise in NF-κB levels after the activation of AKT.

Parallel to this, our results showed that using DMH and γ irradiation for the induction of colon cancer was coupled with abnormal activation of the PI3K/Akt/ mTOR pathway demonstrated with upregulated expression of the PI3K, AKT and mTOR genes confirming the higher proliferative rate of colon cancer cells.

Owing to its role in cellular proliferation in all phases of the cell cycle except G0, it is strongly correlated to the overexpressed AKT due to regulating the tumor cell cycle from G1 to the S phase thus aggravating the proliferation levels of cancerous cells [48]. Li et al. [49] indicated that abnormal activation of AKT and Ki-67 promoted colon cancer development and progression in addition to invasion and metastasis Considering the aforementioned data our results revealed a strong positive expression of Ki-67 in the tissues of rats with colon cancer which agree with the results of Xiao et al. [50] who conducted a noticeable upregulated expression of Ki-67 in mice with colorectal cancer induced by AOM-DSS.

Cancer pathogenesis is associated with dysregulation or inhibition of the cell death signaling pathways including autophagy and apoptosis [51, 52]. Furthermore, Cao et al. [53] indicated the regulating role of autophagy in the development and progression of cancer through the control of the apoptotic pathway. Regarding the activation of the PI3K AKT/signaling the downstream mTOR is activated which is the master regulator of autophagy [54]. Moreover, it directly inhibited autophagy through the phosphorylation of numerous downstream targets [55] like protein complex (ULK1, ATG13 and FIP200) [56] and indirectly through phosphorylation of autophagy/Beclin-1 regulator 1 (AMBRA1), preventing ubiquitination of ULK1 [57]. Kim et al. [58] indicated that the phosphorylation of the ULK1 diminished its interaction with AMPK, the negative regulator of the mTOR, therefore suppressing autophagy. The results of Sun et al. [59] reported that GI cancer progression was linked to the deactivation of the AMPK.

Furthermore, Usman et al. [60] reported lower expression of Beclin1 after its interaction with Bcl-2 (Bcl-2/Beclin1) leading to autophagy suppression. Activation of mTOR and inhibition of autophagy was associated with abnormal accumulation of p62 in cancerous tissues [61]. Coinciding with this, the results of Zhang et al. [62] revealed a dramatic elevation of the p62 expression in colon cancer tissues. In addition to suppressing autophagy, Brech et al. [63] observed that activation of mTOR and AKT inhibited cellular apoptosis via phosphorylation of caspase-3 and caspase-9. Similarly, Su et al. [34] and Sanaei et al. [64] confirmed that AKT hampered the apoptotic process by hindering the activation of the pro-apoptotic proteins concomitant with the upregulation of the ant-apoptotic protein (Bcl-2).

Our results demonstrated a significant overexpression of p62 concomitant with lower expression of AMPK, ATG, LC3II, and Beclin1 in colon tissues of the DMH + IR group. Furthermore, the expression of the pro-apoptotic proteins (Bax) and caspase-3 and caspase-9 were remarkably decreased along with higher levels of the anti-apoptotic protein Bcl-2. These results endorse the abovementioned data and harmonize with the results of Kumar and Agnihotri [47] and Abdel-Wahab et al. [65].

In contrast, owing to the anti-tumor efficiency of 5-FU, the results showed that treatment with5-FUor 5-FUnanogel repressed the TLR2/ NF-κβ pathway manifested by a significant decline in the levels of TLR2 and NF-κβ in colon tissues. Additionally, both downregulated the gene expression of PI3K, AKT, and mTOR consequently suppressing colon cancer proliferation by inhibiting the PI3K/AKT/mTOR pathway. The treatment enhanced the expression of the AMPK as well as the autophagy-related genes LC3II, ATG7 and Beclin-1 and reduced that of P62. Furthermore, the results revealed that 5-FU or 5-FU nanogels triggered apoptosis through an elevation in the expression levels of caspase-3, caspase-9, and Bax coupled with a declined expression of Bcl-2. Moreover, the obtained results showed that the 5-FUnanogel was more effective in suppressing colon cancer proliferation and boosting autophagy and apoptosis relative to that of 5-FU.

Parallel to the obtained results Chen et al. [66] showed that the deactivation of the PI3K/Akt signaling pathway promoted apoptosis in CRC. Moreover, Bu et al. [67] reported that the mTOR dephosphorylation-activated AMPK subsequently enhances autophagy, and apoptosis via Caspase 8-mediated Beclin 1 cleavage in CRC cells [68]. Furthermore, the separation of Beclin-1 from the Bcl-2/Beclin-1 complex increased its levels leading not only to increasing the levels of LC3-B-induced autophagy [58] but also apoptosis in CRC via activation of the caspase-3 through the LC3 autophagy pathway [69]. Additionally, restoring autophagy was associated with abolished expression of p62 and eventually prevented tumor development [70].

## Conclusion

In conclusion, the obtained results exhibited that 5-FU nanogel has an excellent effect rather than the 5-FU which was confirmed by suppressing the proliferation of colon cancer via inhibition of the TLR2/ NF-κβ and PI3K/AKT/mTOR as well as lowering the expression levels of Ki-67. Moreover, it promoted autophagy through the activation of the AMPK and its downstream targets including Beclin-1, ATG, and LC3II along with the downregulation of the p62 gene expression. Additionally, 5-FUnanogel augmented apoptosis by both the intrinsic and extrinsic apoptotic pathways together with repressing the anti-apoptotic protein Bcl-2. Collectively, these data strengthen the therapeutic potential of 5-FU nanogel which can be used as an antitumor product for colon cancer. However, further clinical experiments are needed to assure the exact mechanism.

## Supplementary Information

Additional file1 (DOCX 492 KB)

## Data Availability

All data obtained from this study are included in the current manuscript.
