# Supplementary material for: Antitumor activity of 5-fluorouracil polymeric nanogel synthesized by gamma radiation on a rat model of colon carcinoma: a proposed mechanism
Source: Discov Oncol. 2023 Jul 26;14:138. doi: 10.1007/s12672-023-00733-z (PMC10371941; doi:10.1007/s12672-023-00733-z)
Supplement: Supplementary file 1 — Additional file1 (DOCX 492 KB) [file 12672_2023_733_MOESM1_ESM.docx]

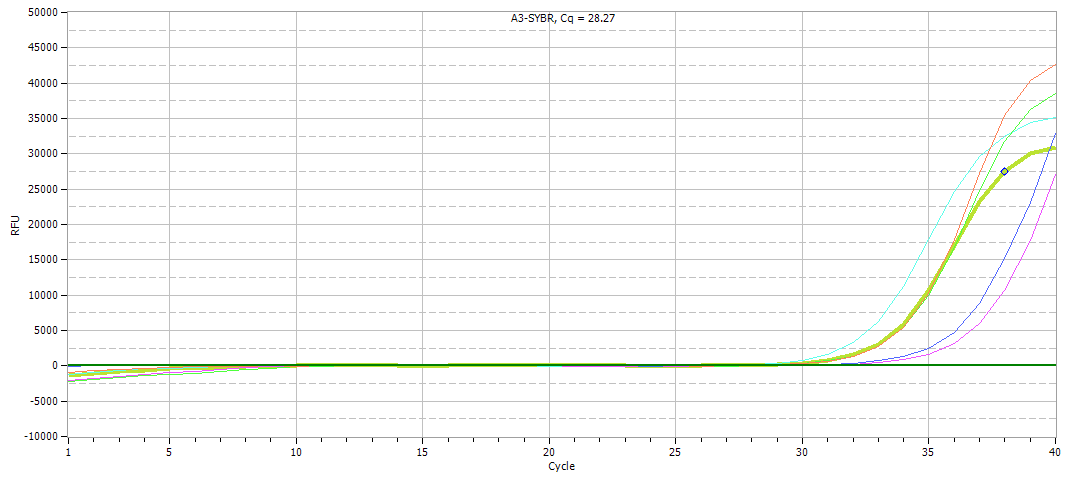


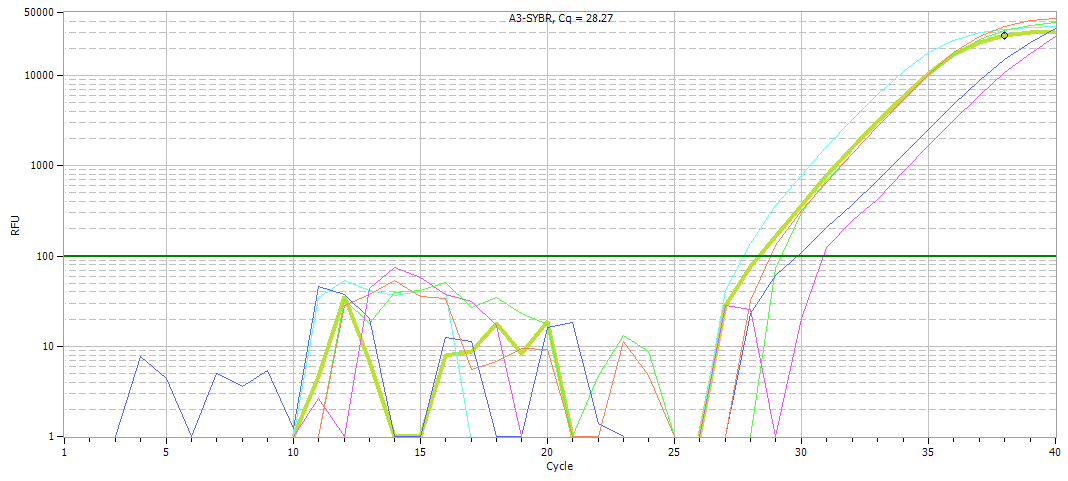


Figure S1: Linear (upper) and log (lower) views of the amplification curves representing the Ct values of *PI3K*.

Supplementary Table (1): Raw data and calculation of the ΔΔCt value of the PI3K gene

| Group | Gene  Aver CT | Delta Ct | Delta delta Ct | Fold change | SEM |
| --- | --- | --- | --- | --- | --- |
| G1 | 28.27 | 3.88 | 0.00 | 1.00 | 0 |
| G2 | 27.6 | 1.87 | -2.01 | 4.03 | 0.21 |
| G3 | 29.12 | 4.15 | 0.27 | 0.83 | 0.1 |
| G4 | 29.79 | 4.18 | 0.30 | 0.81 | 0.11 |
| G5 | 28.7 | 3.80 | -0.08 | 1.06 | 0.14 |
| G6 | 30.76 | 6.62 | 2.74 | 0.15 | 0.01 |


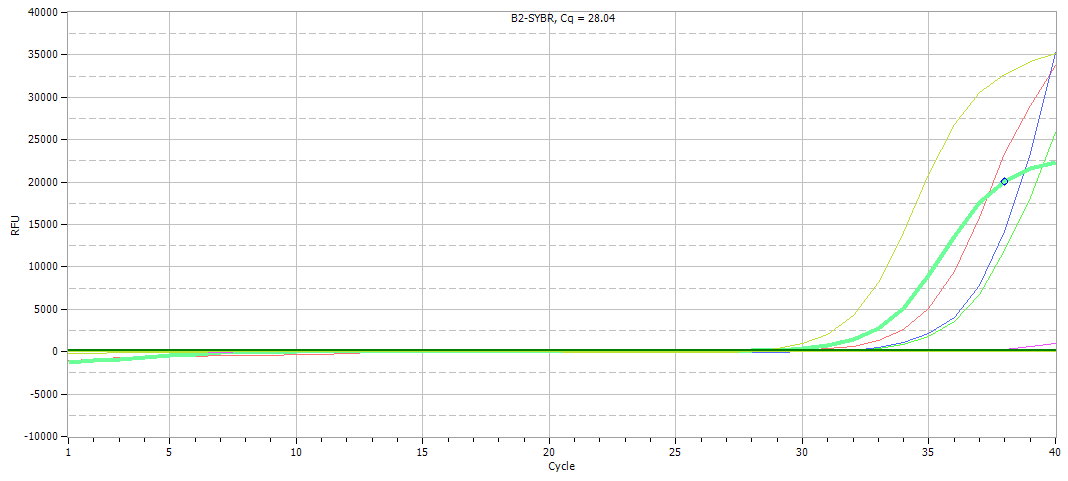


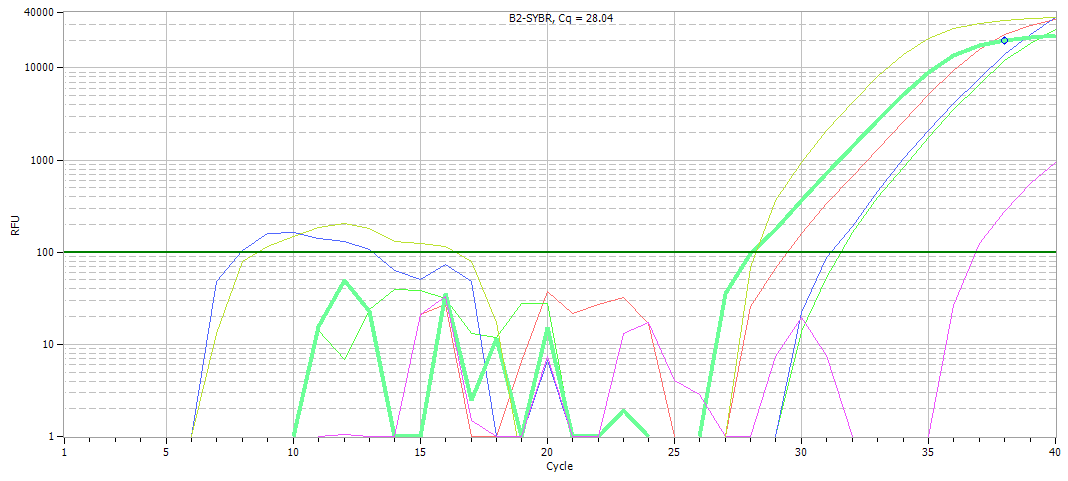


Figure S2: Linear (upper) and log (lower) views of the amplification curves representing the Ct values of *AKT.*

Supplementary Table (2): Raw data and calculation of the ΔΔCt value of the *AKT* gene

| Group | Gene  Aver CT | Delta Ct | Delta delta Ct | Fold change | SEM |
| --- | --- | --- | --- | --- | --- |
| G1 | 31.76 | 7.37 | 0.00 | 1.00 | 0 |
| G2 | 28.04 | 3.31 | -4.06 | 16.68 | 0.82 |
| G3 | 31.13 | 6.86 | -0.51 | 1.42 | 0.17 |
| G4 | 31.42 | 7.11 | -0.26 | 1.20 | 0.19 |
| G5 | 28.11 | 4.02 | -3.35 | 10.20 | 0.47 |
| G6 | 29.36 | 5.22 | -2.15 | 4.44 | 0.26 |


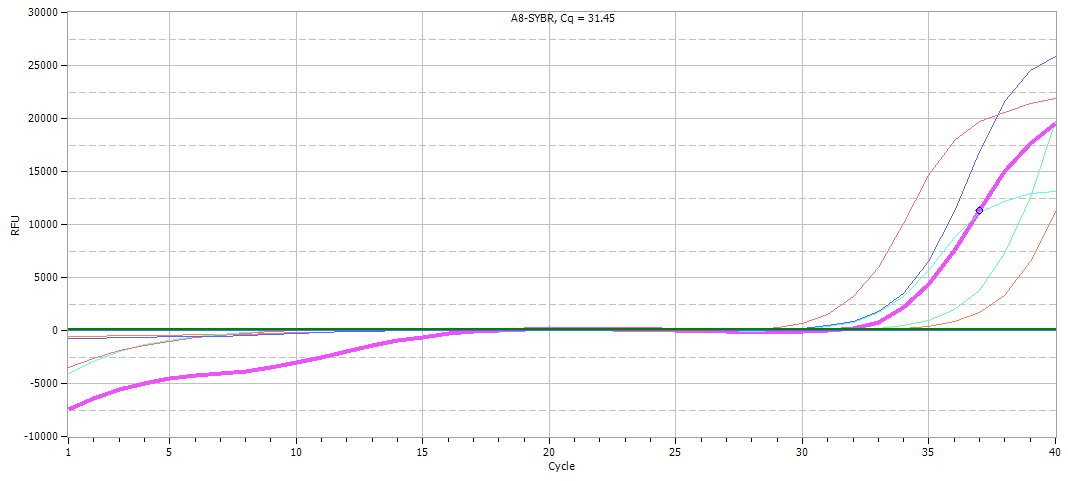


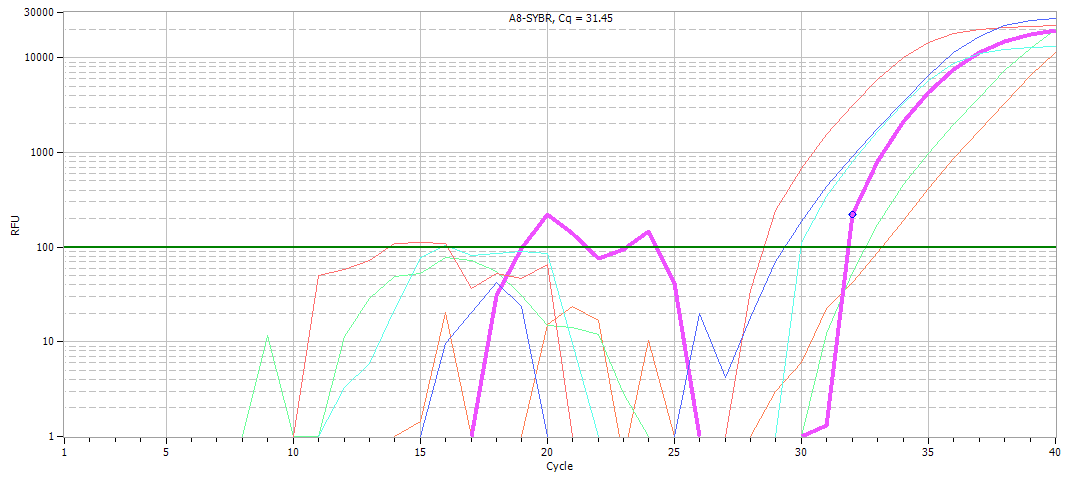


Figure S3: Linear (upper) and log (lower) views of the amplification curves representing the Ct values of *mTOR.*

Supplementary Table (3): Raw data and calculation of the ΔΔCt value of the *mTOR* gene.

| Group | Gene  Aver CT | Delta Ct | Delta delta Ct | Fold change | SEM |
| --- | --- | --- | --- | --- | --- |
| G1 | 33.12 | 8.73 | 0.00 | 1.00 | 0 |
| G2 | 28.31 | 4.18 | -4.55 | 23.43 | 1.05 |
| G3 | 32.39 | 8.12 | -0.61 | 1.53 | 0.47 |
| G4 | 31.45 | 8.02 | -0.71 | 1.64 | 0.52 |
| G5 | 29.25 | 5.16 | -3.57 | 11.88 | 0.6 |
| G6 | 29.91 | 5.77 | -2.96 | 7.78 | 0.39 |


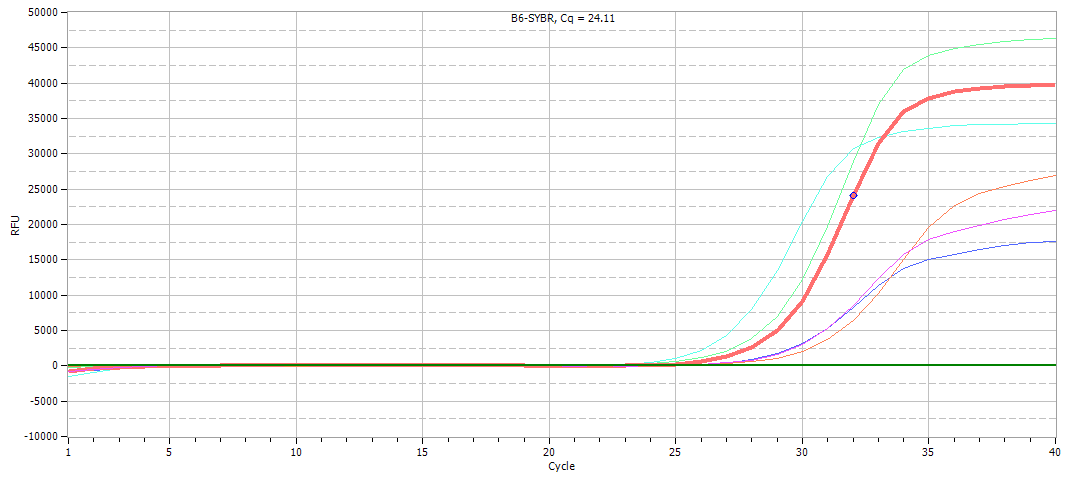


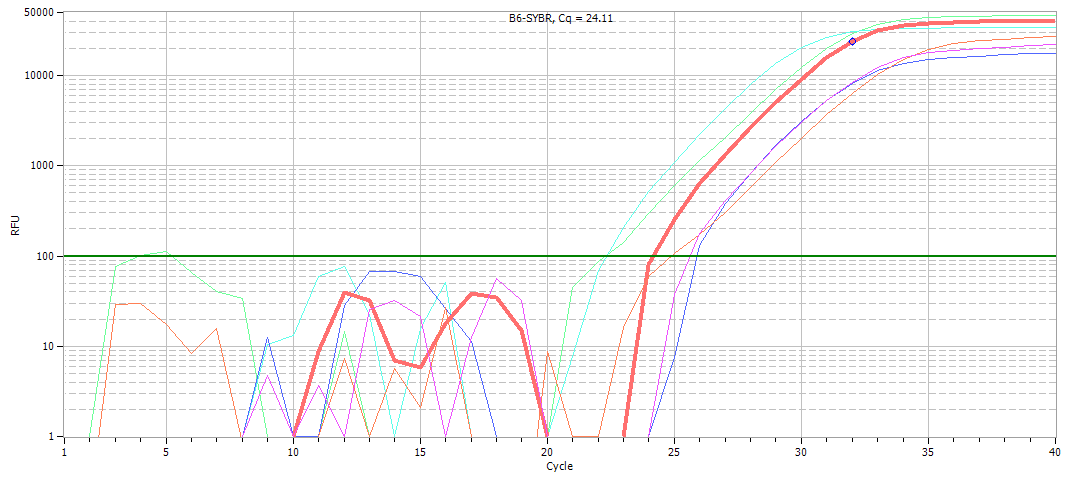


Figure S4: Linear (upper) and log (lower) views of the amplification curves representing the Ct values of *Beclin1.*

Supplementary Table (4): Raw data and calculation of the ΔΔCt value of the *Beclin1* gene

| Group | Gene  Aver CT | Delta Ct | Delta delta Ct | Fold change | SEM |
| --- | --- | --- | --- | --- | --- |
| G1 | 22.22 | -2.17 | 0.00 | 1.00 | 0 |
| G2 | 25.45 | 1.32 | 3.49 | 0.09 | 0.01 |
| G3 | 22.24 | -2.03 | 0.14 | 0.91 | 0.07 |
| G4 | 22.76 | -2.07 | 0.10 | 0.93 | 0.06 |
| G5 | 24.84 | -0.25 | 1.92 | 0.26 | 0.02 |
| G6 | 24.11 | -0.83 | 1.34 | 0.40 | 0.03 |


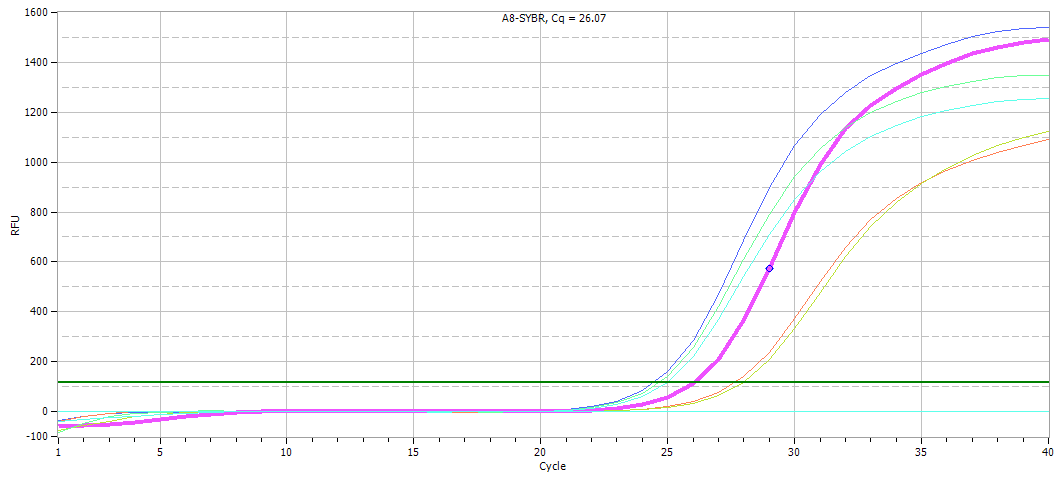


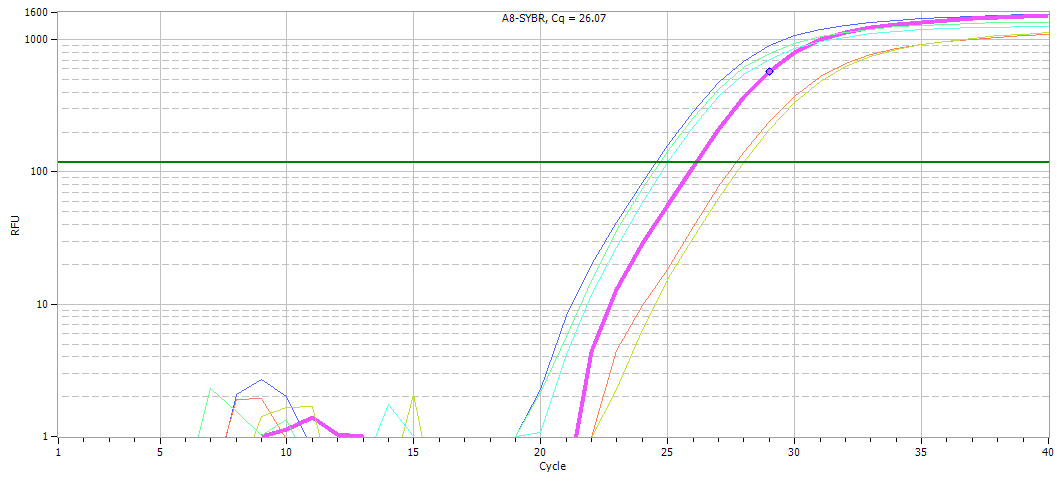


Figure S5: Linear (upper) and log (lower) views of the amplification curves representing the Ct values of *ATG7.*

Supplementary Table (5): Raw data and calculation of the ΔΔCt value of *ATG7* gene

| Group | Gene  Aver CT | Delta Ct | Delta delta Ct | Fold change | SEM |
| --- | --- | --- | --- | --- | --- |
| G1 | 24.45 | 0.06 | 0.00 | 1.00 | 0 |
| G2 | 27.98 | 3.85 | 3.79 | 0.07 | 0.01 |
| G3 | 24.98 | 0.21 | 0.15 | 0.90 | 0.05 |
| G4 | 24.64 | -0.19 | -0.25 | 1.19 | 0.08 |
| G5 | 27.62 | 2.43 | 2.37 | 0.19 | 0.02 |
| G6 | 26.07 | 1.13 | 1.07 | 0.48 | 0.03 |


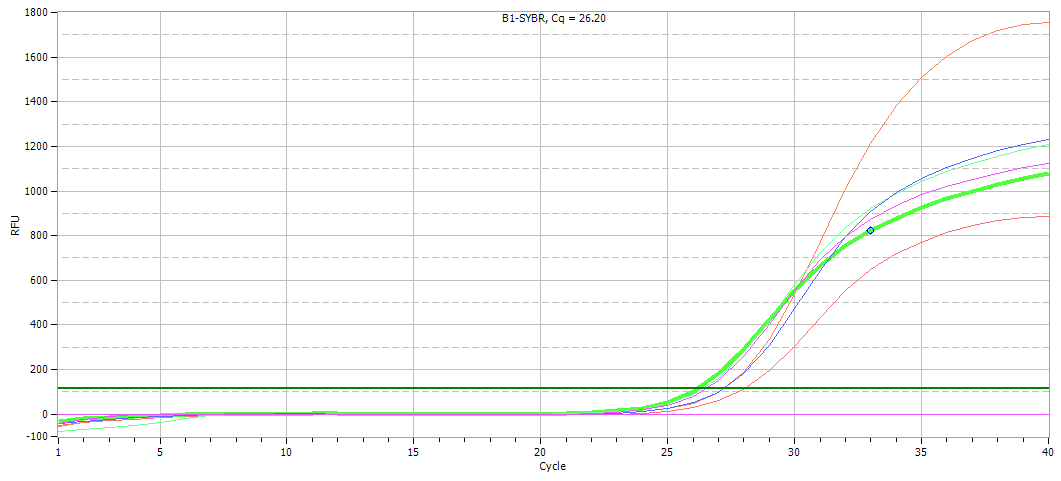

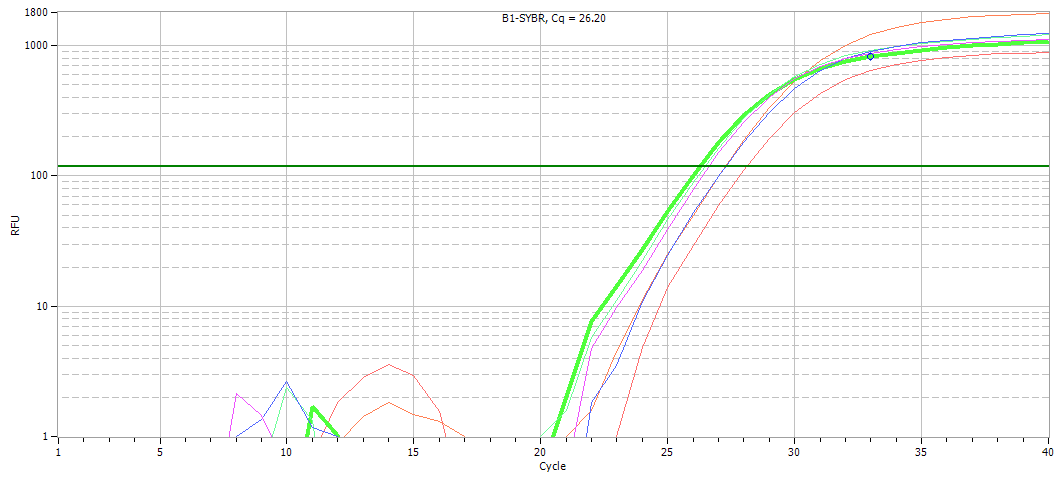


Figure S6: Linear (upper) and log (lower) views of the amplification curves representing the Ct values of *p62.*

Supplementary Table (6): Raw data and calculation of the ΔΔCt value of *p62* gene

| Group | Gene  Aver CT | Delta Ct | Delta delta Ct | Fold change | SEM |
| --- | --- | --- | --- | --- | --- |
| G1 | 28.08 | 3.69 | 0.00 | 1.00 | 0 |
| G2 | 26.2 | 1.47 | -2.22 | 4.66 | 0.26 |
| G3 | 27.21 | 3.14 | -0.55 | 1.46 | 0.17 |
| G4 | 27.22 | 3.19 | -0.50 | 1.41 | 0.16 |
| G5 | 26.39 | 2.10 | -1.59 | 3.01 | 0.14 |
| G6 | 26.53 | 2.39 | -1.30 | 2.46 | 0.09 |


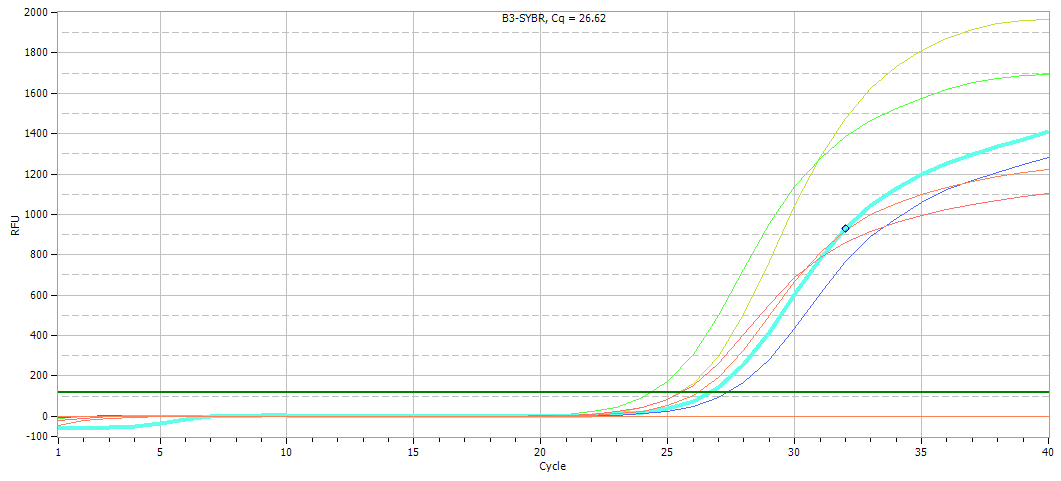


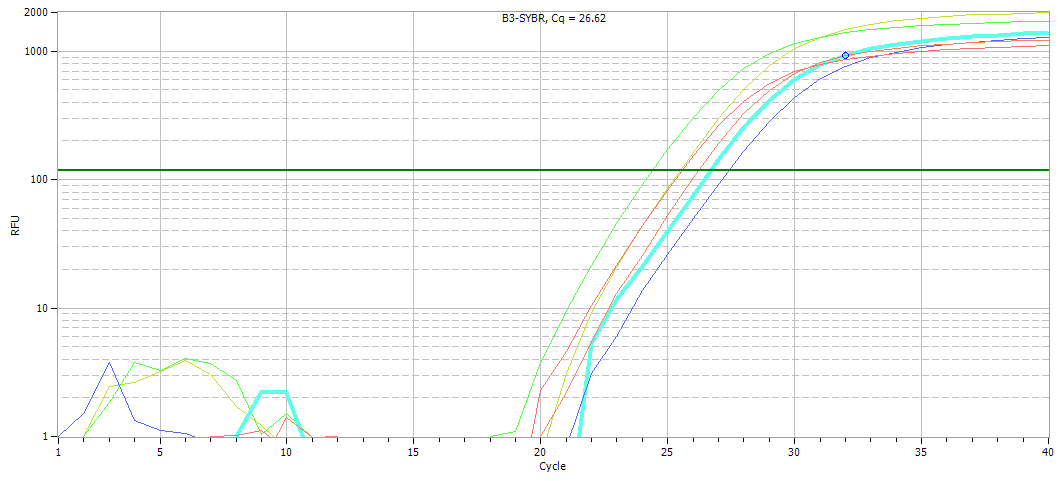


Figure S7: Linear (upper) and log (lower) views of the amplification curves representing the Ct values of the caspase9 *gene.*

Supplementary Table (7): Raw data and calculation of the ΔΔCt value of caspase9 *gene*

| Group | Gene  Aver CT | Delta Ct | Delta delta Ct | Fold change | SEM |
| --- | --- | --- | --- | --- | --- |
| G1 | 25.49 | 1.10 | 0.00 | 1.00 | 0 |
| G2 | 27.35 | 3.12 | 2.02 | 0.25 | 0.01 |
| G3 | 26.15 | 1.58 | 0.48 | 0.72 | 0.05 |
| G4 | 26.62 | 1.79 | 0.69 | 0.62 | 0.04 |
| G5 | 25.42 | 0.43 | -0.67 | 1.59 | 0.09 |
| G6 | 24.32 | -0.52 | -1.62 | 3.07 | 0.13 |


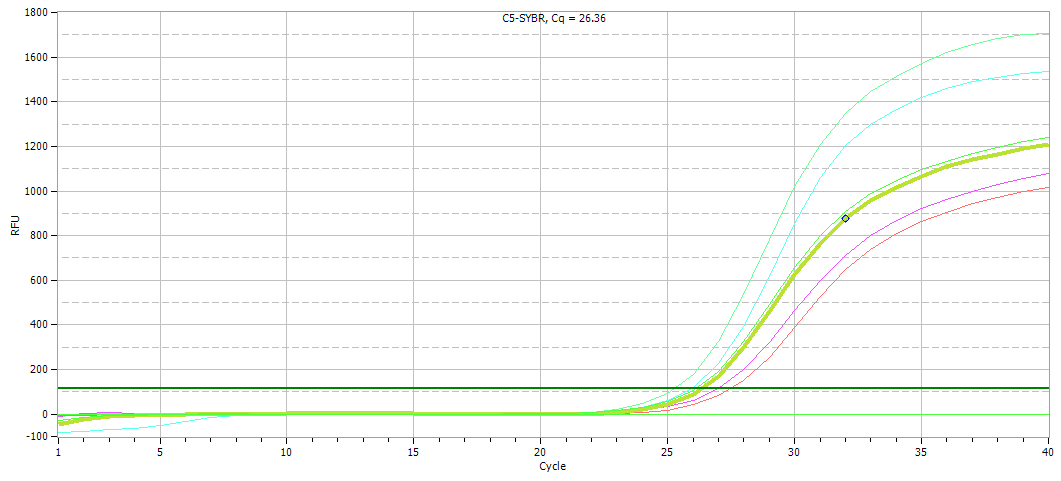


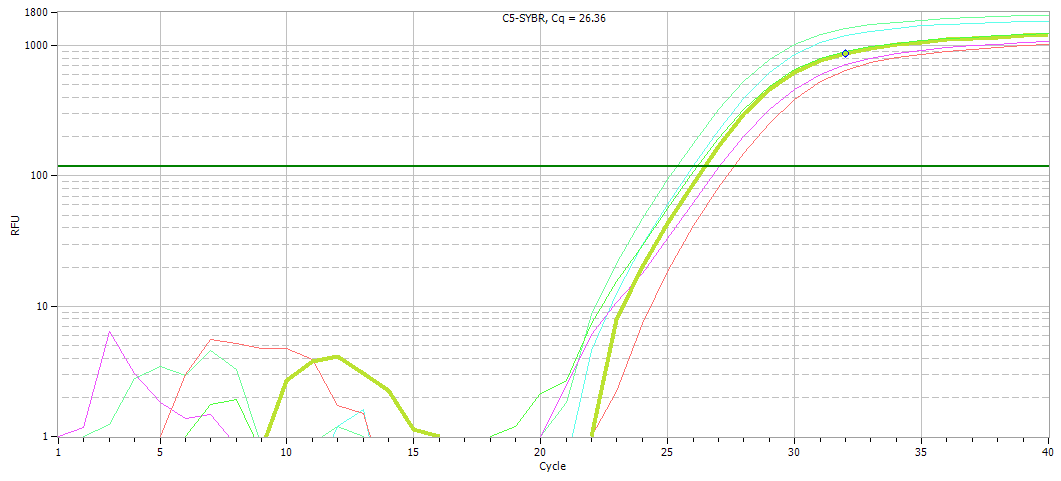


Figure S8: Linear (upper) and log (lower) views of the amplification curves representing the Ct values of the caspase3 *gene.*

Supplementary Table (8): Raw data and calculation of the ΔΔCt value of caspase3 *gene*

| Group | Gene  Aver CT | Delta Ct | Delta delta Ct | Fold change | SEM |
| --- | --- | --- | --- | --- | --- |
| G1 | 27.01 | 2.62 | 0.00 | 1.00 | 0 |
| G2 | 27.52 | 3.29 | 0.67 | 0.63 | 0.03 |
| G3 | 26.36 | 2.29 | -0.33 | 1.26 | 0.12 |
| G4 | 26.12 | 2.09 | -0.53 | 1.44 | 0.15 |
| G5 | 25.96 | 0.97 | -1.65 | 3.14 | 0.14 |
| G6 | 25.27 | 0.43 | -2.19 | 4.56 | 0.18 |


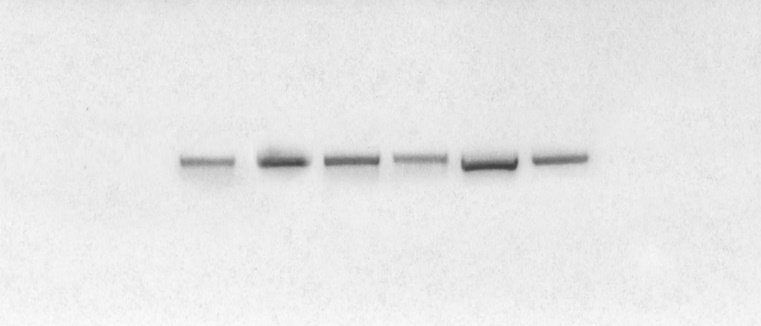


Figure S9: Quantitative western blotting of AMPK protein


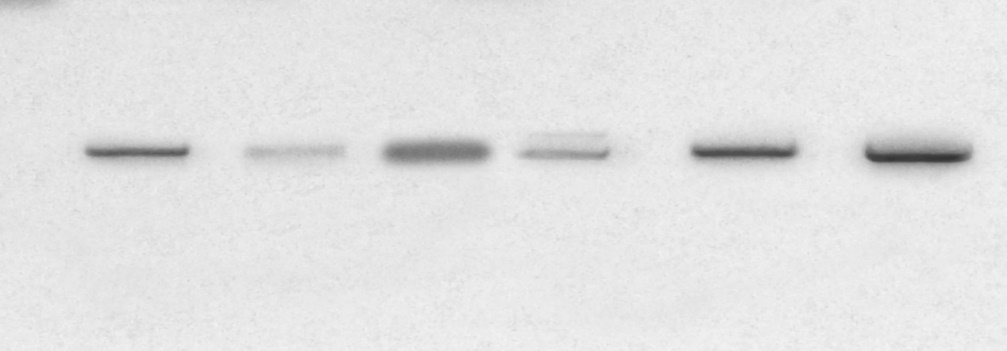


Figure S10: Quantitative western blotting of Bax protein


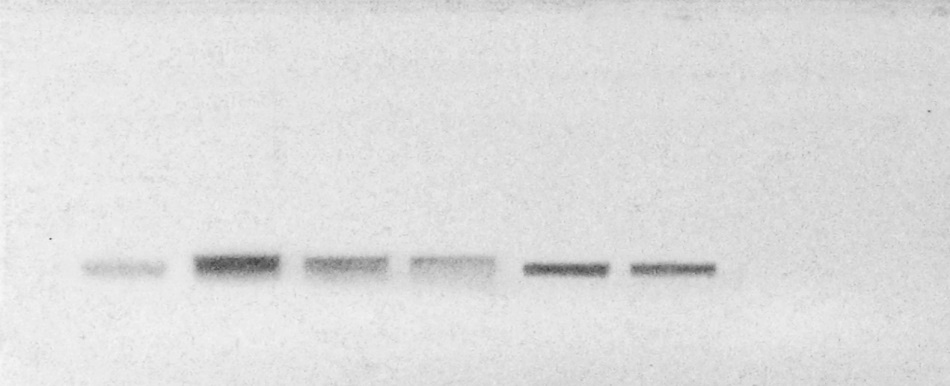


Figure S11: Quantitative western blotting of Bcl2 protein


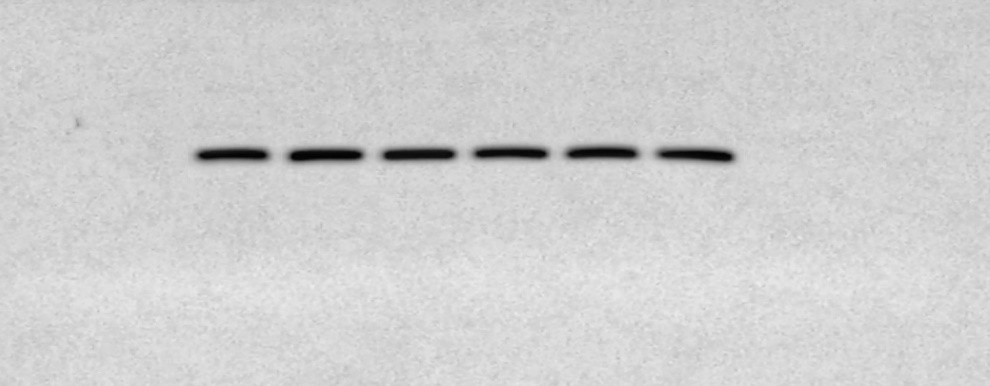


Figure S12: Quantitative western blotting of B-actin protein
